# VANG-1 and PRKL-1 Cooperate to Negatively Regulate Neurite Formation in *Caenorhabditis elegans*

**DOI:** 10.1371/journal.pgen.1002257

**Published:** 2011-09-01

**Authors:** Leticia Sanchez-Alvarez, Jiravat Visanuvimol, Andrea McEwan, Anna Su, Janice H. Imai, Antonio Colavita

**Affiliations:** Ottawa Hospital Research Institute, Neuroscience Program, Heart and Stroke Foundation Centre for Stroke Recovery, University of Ottawa, Ottawa, Canada; University of California San Diego, United States of America

## Abstract

Neuritogenesis is a critical early step in the development and maturation of neurons and neuronal circuits. While extracellular directional cues are known to specify the site and orientation of nascent neurite formation *in vivo*, little is known about the genetic pathways that block inappropriate neurite emergence in order to maintain proper neuronal polarity. Here we report that the *Caenorhabditis elegans* orthologues of *Van Gogh (vang-1)*, *Prickle (prkl-1)*, and *Dishevelled* (*dsh-1*), core components of planar cell polarity (PCP) signaling, are required in a subset of peripheral motor neurons to restrict neurite emergence to a specific organ axis. In loss-of-function mutants, neurons display supernumerary neurites that extend inappropriately along the orthogonal anteroposterior (A/P) body axis. We show that autonomous and non-autonomous gene activities are required early and persistently to inhibit the formation or consolidation of growth cone protrusions directed away from organ precursor cells. Furthermore, *prkl-1* overexpression is sufficient to suppress neurite formation and reorient neuronal polarity in a *vang-1*– and *dsh-1*–dependent manner. Our findings suggest a novel role for a PCP–like pathway in maintaining polarized neuronal morphology by inhibiting neuronal responses to extrinsic or intrinsic cues that would otherwise promote extraneous neurite formation.

## Author Summary

Neurons are among the most morphologically complex cells in the body. Early in development, newly born neurons project one or more processes called neurites that will eventually mature into axons and dendrites. While the genetic determinants that promote neurite emergence along specific trajectories are beginning to be elucidated, the cellular and molecular pathways that prevent inappropriate neurite formation to maintain proper neuronal morphology and prevent superfluous connections are largely unknown. Van Gogh and Prickle dependent-PCP signaling is a well-established regulator of cellular polarity especially along the surface of epithelial cells. In this study, we show that a conserved PCP–like pathway consisting of VANG-1/Van Gogh, PRKL-1/Prickle, and DSH-1/Dishevelled is involved in maintaining the polarized morphology of a subset of neurons in the nematode *C. elegans*. In particular, we show that loss of PRKL-1 results in neurons with too many neurites while PRKL-1 overexpression results in too few neurites. Our findings suggest that mechanisms that specifically block inappropriate neurite formation may be required to ensure proper neuronal connectivity in higher organisms.

## Introduction

Post-mitotic neurons undergo a transition from a more or less symmetrical morphology to a highly polarized one with axonal and dendritic projections that are precisely oriented along body or tissue axes [1], [2]. Neurite emergence is the first overt sign of polarization in newly born neurons followed by differentiation of these neurites into axons and dendrites. While many molecules have been implicated in neuritogenesis and polarity control in cultured neurons, few have been verified by *in vivo* loss-of-function studies [1], [2]. Recently, asymmetrically distributed directional cues such as Netrins and Slits have been shown to polarize protrusive activity in neuronal somas to specify the site of nascent neurite emergence *in vivo* [3], [4]. However, in addition to mechanisms that promote neurite formation, the establishment and maintenance of a polarized morphology also require mechanisms that suppress non-specific neurite growth at all other times. At present, the inhibitory pathways that act *in vivo* to prevent extraneous neurite formation are poorly understood.

Wnt/Frizzled (Wnt/Fz) pathways have been implicated in multiple aspects of post-mitotic neuronal development in animals [5]. Examples in *C. elegans* alone include cell migration [6], axon guidance [7], axo-dendritic polarity [8], and synapse formation [9]. In general, Wnt/Fz signaling pathways can be subdivided into two types, a canonical pathway that involves activation of the transcriptional regulator β-catenin and non-canonical pathways that are β-catenin independent [10]. The Fz/planar cell polarity (Fz/PCP) pathway is a non-canonical Fz pathway that was first identified in *Drosophila* as a key regulator of cell polarity and cell alignment in the plane of the epithelium in eyes, wings, and abdomen [11]–[13]. Subsequent genetic studies in *Drosophila* identified a core group of conserved molecules that are either specific to polarity signaling (Van Gogh/Vang, Prickle, and Flamingo/Fmi/Celsr) or common to all Fz pathways (Dishevelled/Dsh/Dvl) [11]–[13]. In vertebrates, Fz/PCP signaling has been implicated in a diverse set of polarized cellular outcomes such as the ordered alignment of sensory hairs of the inner ear [14], oriented cell division [15], and directed convergent extension movements during gastrulation and neurulation [16]–[18]. The recent finding that Frizzled3, Vangl2/Van Gogh, and Celsr3/Flamingo mutants share similar axon guidance defects in mice also implicate Fz/PCP signaling in growth cone navigation [19], [20].

**Figure 1:**
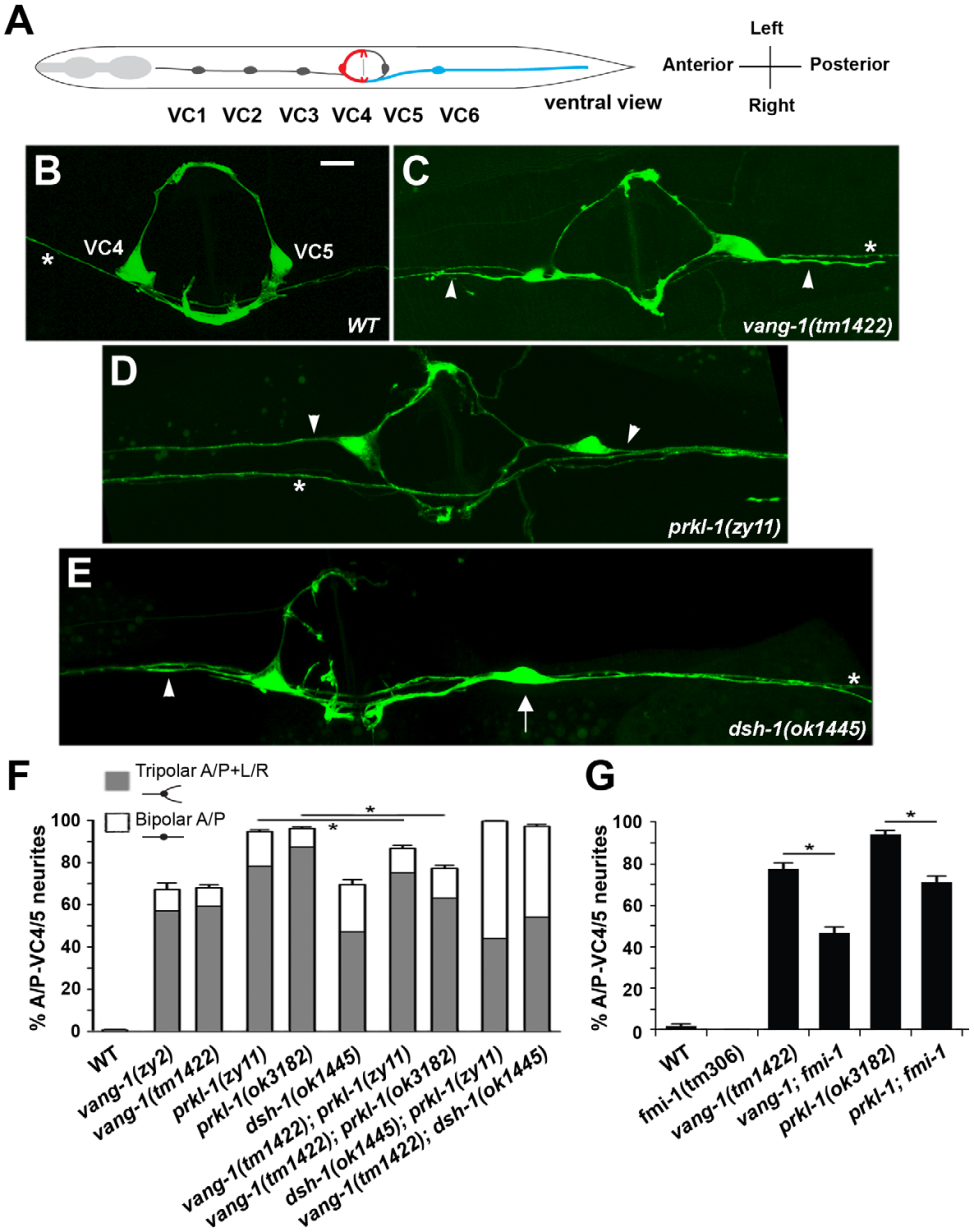
A PCP-like pathway that includes *vang-1*, *prkl-1*, and *dsh-1* restricts nascent neurite formation to a specific tissue axis. (A) Adult worm schematic (ventral view) showing VC1–6 position and neurite placement along the A/P body or L/R vulval axes. VC4 and VC5 neurons extend neurites laterally (L/R) along the vulval epithelium, whereas VC1–3 and VC6 extend neurites along the A/P body axis. Representative images of adult VC4 and VC5 neurons in wild-type (B) and displaying supernumerary A/P-directed neurites (arrowheads) in *vang-1* (C), *prkl-1* (D), and *dsh-1* (E) mutants. While most neurons in PCP mutants display supernumerary neurites (resulting in tripolar morphologies), some like the VC5 neuron (arrow) in (E) display an atypical A/P-bipolar morphology. (B–E) Asterisks mark non-VC4 and VC5 labeled processes in the ventral cord. Scale bar, 10 µm. (F) Quantification of VC4/5 polarity defects in *vang-1*, *prkl-1*, and *dsh-1* single and double mutants, n>250. (G) An *fmi-1* loss-of-function allele suppresses the VC4/5 polarity defects of *vang-1* and *prkl-1* alleles, n>100. *cyIs4* was used to label VC4 and VC5 neurons in (B–F) and *cyIs1* in (G). Error bars in (F) and (G) represent standard error of proportion, *p<0.01, χ^2^.

To identify determinants of neuronal polarity in *C. elegans*, we utilized the bipolar VC neurons, a set of six peripheral motor neurons that mediate egg-laying [21]. The VC neurons are an ideal polarity model as they display stereotypical differences in the orientation and extension of process outgrowth relative to the A/P axis and proximity to target structures. VC1–3 and VC6 project processes along the A/P axis, whereas the processes of VC4 and VC5 project along the orthogonal left-right (L/R) axis generated by the developing vulva, an intermediate target tissue during organ innervation (Figure 1A and Figure S1). The lateral placement of VC4 and VC5 processes ensures that connections are made with HSN neuronal and vm2 vulval muscle targets located on the left and right sides of the vulva [22]. The directional cues that orient VC process growth bidirectionally along the A/P axis or toward vulval cells are not known. However, in animals in which the vulva has been physically or genetically ablated, the processes of VC4 and VC5, like those of VC1–3 and VC6, extend along the A/P axis [23], suggesting that bidirectional A/P growth constitutes the default polarity in the absence of vulval-derived cues.

In this study, we show that the worm orthologues of the core PCP molecules Van Gogh (VANG-1), Prickle (PRKL-1), and Dishevelled (DSH-1) are required to ensure that neurite emergence in VC4 and VC5 occurs exclusively along the vulval organ axis during innervation. Loss of these molecules result in the emergence of ectopic VC4 and VC5 neurites along the A/P axis directed away from vulval cells. We show that mature neurons are more severely affected than developing ones suggesting a primary fault in polarity maintenance. We also show that neurite growth inhibition involves autonomous and non-autonomous components and is required in a persistent manner to maintain neuronal morphology. PRKL-1 plays a key role in this process as *prkl-1* overexpression is sufficient to suppress neurite formation and reorient VC polarity along the A/P axis in a *vang-1* and *dsh-1*-dependent manner. These findings demonstrate a novel role for PCP-like signaling in blocking inappropriate neurite formation to maintain the polarity of initial neurite emergence in neurons.

## Results

### *vang-1* is involved in specifying the polarity of VC neurons along the A/P axis

To begin to investigate how the polarity of VC neurite outgrowth is specified we characterized *zy2*, a mutation that was identified serendipitously in a genetic screen for VC neurite branching defects (A. C. and M. Tessier-Lavigne, unpublished results). As VC processes, like those of many *C. elegans* neurons, display a mixed axonal and dendritic identity along their lengths [24], they will herein be referred to as neurites. In *zy2* mutants, VC4 and VC5, visualized using the *Pcat-1::GFP* transgene *cyIs4* [25], project inappropriate neurites along the A/P axis (Figure 1B and 1C). These extraneous neurites result in highly penetrant tripolar VC4 and VC5 morphologies in which two neurites project normally along the L/R axis of the vulva and a third extends inappropriately along the A/P axis away from the vulva. These mutants also displayed less penetrant A/P-bipolar VC4 and VC5 morphologies like those of VC1–3 and VC6 (Figure 1F). All other aspects of VC morphology including terminal arborizations [26] are normal in *zy2* mutants. High resolution genetic mapping followed by sequencing of candidate loci (data not shown) revealed *zy2* to be a premature stop within *vang-1* (Figure S2), the sole worm orthologue of Van Gogh/Strabismus, a core component of the Fz/PCP pathway in vertebrates and invertebrates [27]. *tm1422*, a *vang-1* deletion and putative null allele [27], displayed similar VC phenotypes.

### A conserved PCP pathway inhibits VC4 and VC5 neurite formation along the A/P axis

To determine if the identification of *vang-1* implicated a conserved planar polarity pathway in regulating VC polarity, we examined VC4 and VC5 morphology in other candidate PCP mutants. In addition to Van Gogh, other core PCP mediators include the cytoplasmic proteins Prickle and Dishevelled (Dsh) and the cell-surface proteins Frizzled (Fz) and Flamingo [11], [12], [13]. The worm genome contains a single Prickle (*prkl-1*) and Flamingo (*fmi-1*) and several Fz (*lin-17*, *mig-1*, *cfz-2*, and *mom-5*) and Dsh (*dsh-1*, *dsh-2*, and *mig-5*) orthologues (WormBase, http://www.wormbase.org). As Fz and Dsh molecules are common to both canonical and non-canonical Wnt/Fz signaling pathways and some of these play essential roles during early embryonic and vulval development [28], we limited our analysis to homozygous viable mutants with grossly normal vulval morphology (Table S1).

Of the core PCP components mentioned above, only Van Gogh and Prickle are specific to Fz/PCP signaling pathways. *vang-1* has been implicated in intestinal tube formation [27] and the orientation of vulval precursor cell (VPC) polarity [29]. *prkl-1* function in *C. elegans* has not been previously characterized. PRKL-1 exists as two isoforms, a long isoform (PRKL-1A) containing an N-terminal PET domain, three LIM domains, and a C-terminal CAAX prenylation motif and a short isoform (PRKL-1B) lacking the PET domain. Blast searches reveal several PET and LIM containing proteins, but only PRKL-1A displays a domain organization similar to the Prickle orthologues in *Drosophila*, fish and mammals (Figure S3). RNAi-mediated knock-down of *prkl-1* produced ectopic A/P-directed VC4 and VC5 neurites similar to those displayed by *vang-1* mutants (data not shown). We subsequently confirmed this finding in *prkl-1(zy11)*, a premature stop allele, retrieved in a non-complementation screen over a chromosomal deficiency *(nDf41)* that deletes the *prkl-1* locus and *prkl-1(ok3182)*, a deletion and candidate null allele generated by the *C. elegans* Gene Knockout Consortium. VC defects were not detected in *mig-1/Fz* and *cfz-2/Fz* single or double mutant combinations and in maternally rescued *mom-5/Fz* mutants (Table S1). Loss of *cat-1* promoter activity from *cyIs4[Pcat-1::GFP]* (Table S1), our only VC4 and VC5 specific reporter gene, and secondary neuronal morphology defects caused by vulval abnormalities in *lin-17* mutants and combinations with other Fz mutants [29], [30], precluded an unambiguous assessment of Fz function in VC polarity. Overall, *vang-1*, *prkl-1*, and *dsh-1* mutants share strikingly similar VC4 and VC5 polarity defects, although those of *prkl-1* were quantitatively more severe (Figure 1D–1F). *dsh-1* mutants also displayed a slightly greater proportion of bipolar VC4 and VC5 neurons with A/P-oriented neurites like those of VC1–3 and VC6 (Figure 1E and 1F).

Interestingly, VC polarity defects were not detected in *fmi-1(tm306)*, a strong loss-of-function (lf) Flamingo mutant (A. Steimel and H. Hutter, personal communication) (Figure 1G). This contrasted sharply with a role for Flamingo in most, if not all, currently known manifestations of PCP mediated through Fz/PCP signaling [11]–[13]. We therefore further assessed the involvement of FMI-1 in VC polarity by examining worms bearing simultaneous losses in both *fmi-1* and *vang-1* or *prkl-1*. Loss of *fmi-1* in a *vang-1(lf)* or *prkl-1(lf)* background resulted in a mild but significant suppression of VC polarity defects (Figure 1G). This finding suggests that FMI-1 normally promotes neurite growth in VC4 and VC5, a role which is revealed only when the normally neurite inhibitory roles of VANG-1 or PRKL-1 are removed.

Excluding ectopic neurites, we did not observe additional VC4 and VC5 morphology, branching, or pathfinding defects in *vang-1*, *prkl-1*, or *dsh-1* mutants consistent with a specific dysregulation of neurite formation and not general growth cone motility or axon guidance. A survey of major longitudinal and commissural axon tracts also failed to reveal additional wiring defects in *vang-1* and *prkl-1* mutants (data not shown). This survey included the PLM mechanosensory neurons which have been shown to require a *lin-44*/Wnt-*lin-17*/Fz pathway to specify the A/P orientation of axon and dendrite polarity [8]. In *lin-44* or *lin-17* mutants, PLM polarity is reversed with axon and dendrites projecting in opposite directions. In addition to normal PLM polarity in *vang-1* and *prkl-1* mutants, the penetrance of *lin-17* polarity defects were also not affected in double mutants with *vang-1(lf)* or *prkl-1(lf)* (data not shown). Although it is not known if Fz genes act in VC polarity, this finding suggests that distinct Fz pathways regulate neuronal polarity in different subtypes of neurons in *C. elegans*.

### VC polarity defects do not result from defects in vulval cell induction or polarity

Our findings suggest that a PCP-like pathway that includes VANG-1 and PRKL-1 acts to block the emergence of VC4 and VC5 neurites along the A/P axis. Alternatively, we considered the possibility that the polarity phenotype was a secondary consequence of a cell fate change in VC4 and VC5 or VPCs that resulted in the acquisition of VC1–3 and VC6-like characteristics such as A/P-directed neurite growth. Previous work has shown that VC4 and VC5 extend neurites bidirectionally along the A/P axis in mutants affecting VPC induction, such as those in the RAS-MAP kinase pathway [23]. Furthermore, in addition to roles for Wnt/Fz signaling in VPC fate specification [30], distinct Wnt pathways involving *lin-17*/Fz, *lin-18*/Ryk, and a *vang-1* pathway that includes *egl-20*/Wnt and *cam-1*/Ror regulate VPC polarity [29]. However, several observations suggest that VC polarity phenotypes do not arise from cell fate or VPC polarity defects. First, *vang-1*, *prkl-1*, and *dsh-1* single mutants display normal vulval morphology suggesting normal vulval cell induction and polarity (Figure S4). Second, we did not find strong VC polarity defects in *lin-18*, *cam-1*, or *egl-20* VPC polarity mutants (Table S1). Indeed, while approximately 36% of P7.p VPCs display reversed polarity in *lin-18(e620)* mutants [29], VC4 and VC5 polarity in these animals is largely unaffected (Table S1). Finally, promoter activity from the *cat-1*/vesicular monoamine transporter gene *(Pcat-1::GFP)*, a molecular marker that is specifically expressed in VC4 and VC5 but not VC1–3 and VC6 [31], is largely unaffected in *vang-1* and *prkl-1* mutants (Table S1). This contrasts sharply with a prominent role for a β-catenin-dependent *lin-44*/Wnt-*lin-17*/Fz pathway in promoting *cat-1* gene expression in VC4 and VC5 (Table S1).

### *vang-1* and *prkl-1* act in the same pathway to inhibit neurite growth

The similar polarity defects displayed by *vang-1*, *prkl-1*, and *dsh-1* mutants suggest that these genes act in a common pathway to block neurite growth. To test this notion, we examined double mutant combinations, reasoning that if two genes act in the same pathway, then the resulting defects should not be more severe than those of the strongest single mutant. The molecular lesions in *vang-1(tm1422)*, *prkl-1(zy11, ok3182)*, and *dsh-1(ok1445)* are predicted to generate early terminations in gene products and are thus likely to generate strong reduction or loss of gene function (Figure S2). We found that the penetrance of A/P-oriented VC4 and VC5 neurites in *vang-1; prkl-1* double mutants was not more severe than *prkl-1* single mutants consistent with *vang-1* and *prkl-1* acting in the same pathway. Indeed, we found a small but significant (P>0.01) suppression of the polarity phenotype in these animals compared to loss of *prkl-1* alone (Figure 1F). In contrast, the proportion of bipolar VC4 and VC5 neurons displaying an A/P-bidirectional neurite orientation increased in *vang-1; dsh-1* and *dsh-1; prkl-1* double mutants compared to single mutants (Figure 1F). However, we found that these double mutants, unlike single mutants or *vang-1; prkl-1* double mutants, displayed vulval morphology defects (Figure S4), suggesting that part or all of this increase may be secondary to underlying defects in VPC specification. Alternatively, in addition to blocking A/P-directed neurite growth, *vang-1* and *prkl-1* may also act in parallel with a *dsh-1*-containing pathway to inhibit default A/P polarity and/or promote neurite growth along the L/R vulval axis. Therefore, it is not clear at this time if *dsh-1* acts in the same pathway as *vang-1* and *prkl-1* or in a parallel pathway to block A/P-directed neurite growth in VC4 and VC5.

### PCP genes are expressed in VC neurons and vulval epithelial cells during neurite extension

**Figure 2:**
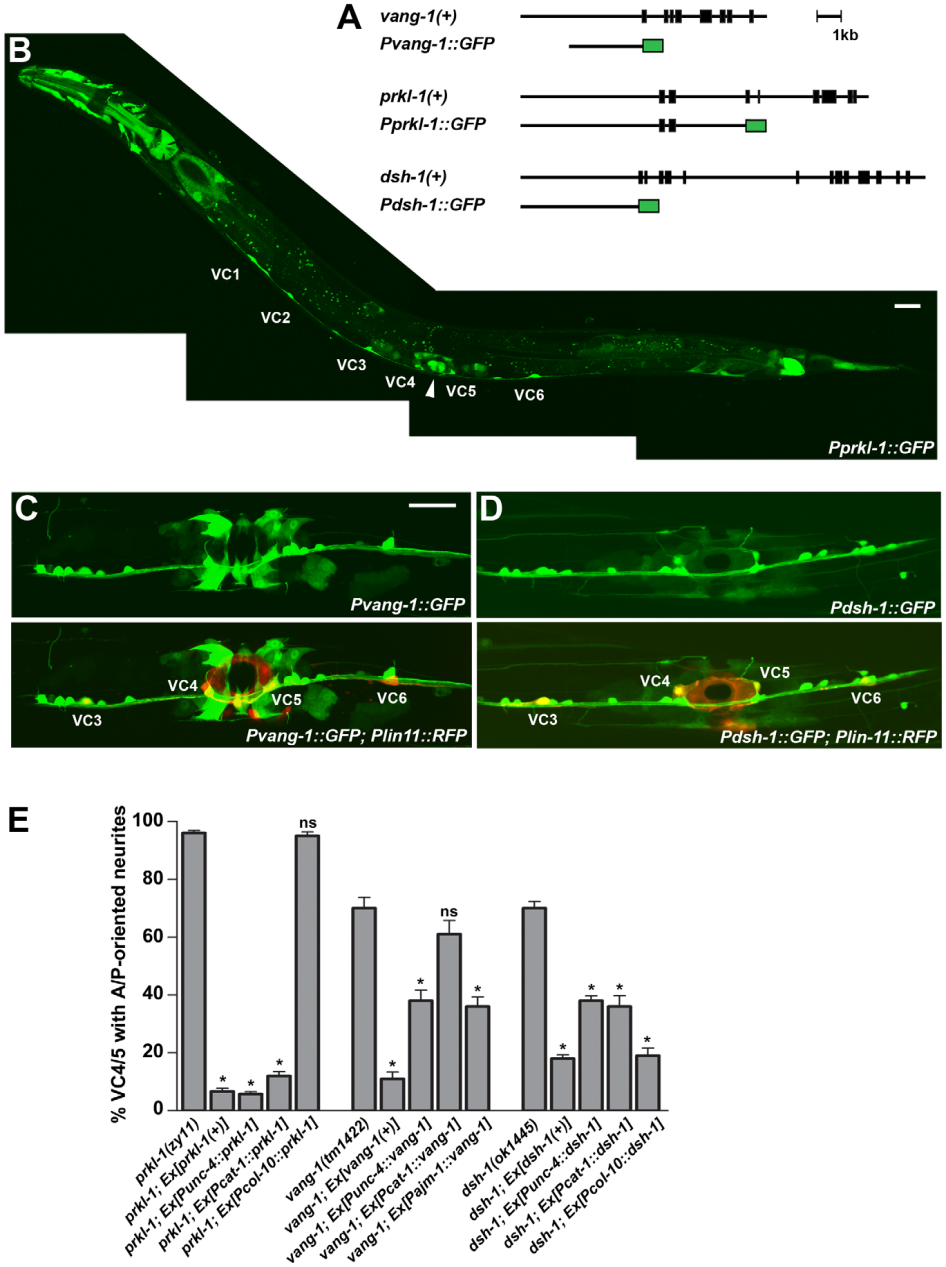
PCP genes act autonomously in VC4 and VC5 and non-autonomously from epithelial cells to inhibit neurite growth. (A) Schematic representation of genomic rescuing constructs and GFP transcriptional reporters used in this study. (B–D) *prkl-1* (B), *vang-1* (C), and *dsh-1* (D) transcriptional activity (GFP) is present in VC neurons during the period of VC neurite extension (L4 stage) and in vulval cells (arrowhead in B). Expression is also seen in a subset of ventral cord neurons and uterine cells. Due to widespread expression of *vang-1* and *dsh-1* in neurons, the VC1–6 and vulval cell reporter *Plin-11::RFP* was used to unambiguously identify VC neurons (yellow in merged image). Scale bars, 20 µm. (E) Cell-specific rescue experiments using functional GFP-tagged proteins suggest that *prkl-1* acts autonomously in VC neurons, whereas *vang-1* and *dsh-1* act both autonomously in VC neurons and non-autonomously to restrict neurite emergence. Representative transgenic lines are shown, but at least two independent lines were examined per experiment. All lines carry a *cyIs4* reporter. Error bars represent standard error of proportion; n>100. *p<0.001, χ^2^ (compared to non-transgene expressing mutants).

We next made transgenic animals carrying GFP transcriptional fusions to determine *vang-1*, *prkl-1*, and *dsh-1* expression patterns during the period of neurite extension and pathfinding in L4. *Pvang-1::GFP* and *Pdsh-1::GFP* reporters each contain at least 3 kb of promoter region upstream of the ATG start site fused to GFP. The *Pprkl-1::GFP* reporter contains a 9.2 kb genomic region fused in-frame to GFP at exon 3 (Figure 2A). In L4 larvae, *vang-1* and *dsh-1* promoter activity was detected in many head and ventral cord neurons, some bilaterally located mid-body neurons including the HSNs, as well as non-neuronal tissue such as somatic gonad cells, uterine cells, vulval muscle and vulval epithelial cells (Figure 2C and 2D). Co-expression with the VC1–6 specific reporter *Plin-11::RFP* [32] revealed that the subset of ventral cord neurons expressing *vang-1* and *dsh-1* includes all six VC neurons. Interestingly, in contrast to the more widespread neuronal expression of *vang-1* and *dsh-1* during L4, *Pprkl-1::GFP* was only consistently detected in VC1–6 and the HSN neurons and a small subset of neurons in the head and ventral nerve cord (Figure 2B). In non-neuronal tissue, *prkl-1* promoter activity was detected in somatic gonad cells, uterine cells, distal tip cells, and vulval cells (Figure 2B). These findings indicate that *vang-1*, *prkl-1*, and *dsh-1* are expressed in the VC neurons as well as the vulval guidepost cells that act as VC intermediate target cells during pathfinding. We also made GFP-tagged genomic translational fusions but these constructs (*Ex[prkl-1(+)]*, *Ex[vang-1(+)]*, and *Ex[dsh-1(+)]*) disrupted embryonic development when over-expressed from transgenic arrays. At lower expression levels, these constructs could restore normal VC polarity in PCP mutants (Figure 2E) and, despite generally weaker fluorescence, displayed the same VC and vulval cell expression patterns observed with transcriptional reporters.

### PCP genes act autonomously and non-autonomously to inhibit neurite formation

To determine if *vang-1*, *prkl-1*, or *dsh-1* act cell autonomously to regulate neuronal polarity, we assessed the ability of extrachromosomal arrays bearing genes expressed from either neuronal or epithelial specific promoters to restore normal VC4 and VC5 polarity in mutant animals. Neuronal specific expression was realized using the *unc-4* promoter which is active transiently in a subset of ventral cord neurons in the early larva and continuously in VC1–6 beginning in L3 [33] and the *cat-1* promoter which is active in VC4 and VC5 beginning in early L4 [31]. Epithelial specific expression was obtained using the *col-10* or *ajm-1* promoters which are active in all epithelial cells including vulval cells [34], [35]. In each case, proteins were also N-terminally (VANG-1 and PRKL-1) or C-terminally (DSH-1) fused to GFP to confirm gene expression *in vivo*. We found that *prkl-1* expressed in neurons but not epithelial cells restored normal polarity in mutant animals similar to that achieved using a genomic *prkl-1* construct (Figure 2E). In contrast, both neuronal and epithelial specific-expression of *vang-1* and *dsh-1* rescued polarity phenotypes, although with the exception of *Pcol-10::dsh-1*, less strongly than the genomic constructs (Figure 2E). Interestingly, *Pcat-1*::*vang-1* expression did not rescue *vang-1(lf)* polarity defects. This may be explained by the later onset of *Pcat-1* expression in VC4 and VC5 compared to *Punc-4* and may imply that VANG-1 is required at an earlier stage than PRKL-1 or DSH-1 to regulate polarity. Combined, these data suggest that *prkl-1* acts autonomously and that *vang-1* and *dsh-1* act both autonomously and non-autonomously to inhibit inappropriate VC4 and VC5 neurite growth. These findings mirror those in other PCP models that involve prominent roles for both cell autonomous and non-cell autonomous gene activity [12], [36]. In addition to VC1–6, the *unc-4* promoter is also expressed at early stages in the DA and VA motor neurons that mediate backward movement [33]. Interestingly, *Punc-4::GFP::prkl-1* expressing lines, but not *Punc-4* driven *vang-1* and *dsh-1* lines, displayed a variable but strong inability to move backwards (data not shown), suggesting a specific ability of ectopic *prkl-1* expression to compromise the function of the DA and/or VA neurons.

### PCP-like signaling maintains VC4 and VC5 polarity after a bipolar to unipolar reorientation of leading edge protrusions along the A/P axis

**Figure 3:**
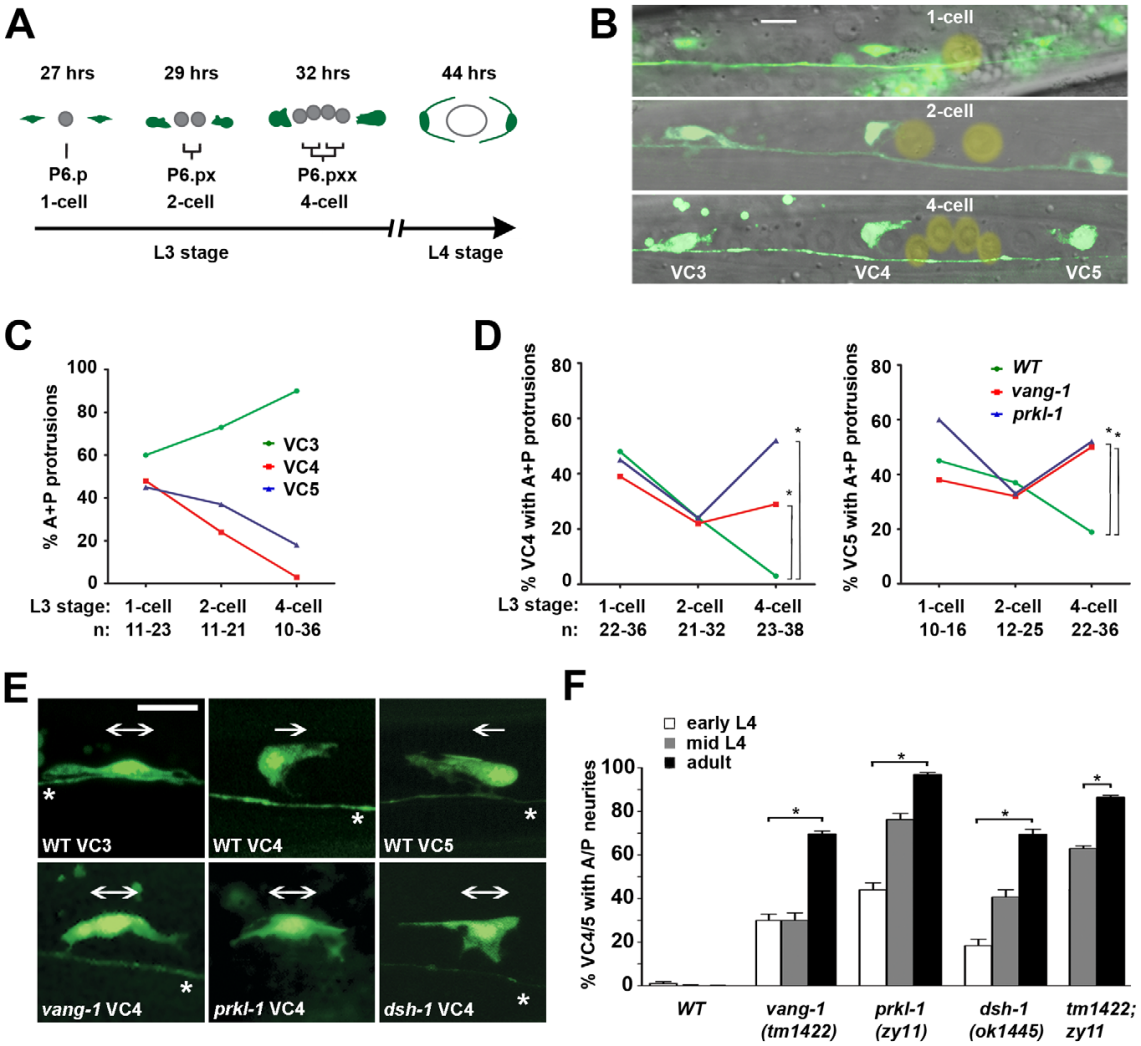
PCP-like signaling maintains VC4 and VC5 polarity after a bipolar to unipolar reorientation along the A/P axis. (A) Simplified schematic showing a timeline (hours post-hatching) of VC4 and VC5 neuritogenesis and vulval organogenesis from early L3 to mid-L4 (see also Figure S1). Division of the P6.p vulval precursor cell (VPC) was used as milestones for staging during L3. (B) Representative merged fluorescence and DIC images showing the morphology of VC3, VC4, and VC5 at the 1-cell, 2-cell and 4-cell P6.p VPC stages. P6.p and its descendents are marked with a yellow overlay. (C and D) From the 1-cell to the 4-cell stage in wild-type animals, the proportion of neurons displaying an A/P-elongated bipolar morphology progressively increases for VC3 and decreases for VC4 and VC5 (C). In PCP mutants, a significant proportion of VC4 and VC5 neurons display an A/P bipolar morphology compared to wild-type by the 4-cell stage (D), suggesting that PCP signaling is required early during neuritogenesis to restrict neurite emergence. *p<0.05, Fisher's Exact test. (E) Representative micrographs at the 4-cell stage reveal that VC4 and VC5, normally polarized unidirectionally toward the vulval axis of symmetry (unipolar arrows), resemble the A/P bipolar morphology of VC3 in *vang-1*, *prkl-1* and *dsh-1* mutants (bipolar arrows). (F) In PCP mutants, VC4 and VC5 display a gradual loss in polarity during development suggesting that PCP signaling is required to maintain polarized morphology. Error bars represent standard error of proportion; n>100. *p<0.001, χ^2^. VC neurons in B and D visualized using a *cyIs3[Punc-4::GFP]* reporter. Scale bar, 10 µm.

VC neurons are born during L1 with neuritogenesis and neurite outgrowth delayed until the late L3 stage after the onset of vulval development [22] (Figure 3A and Figure S1). Vulval organogenesis begins with the induction and subsequent divisions of three vulval precursor cells (P5.p, P6.p, P7.p) followed by morphogenetic movements to generate the mature vulva [37]. To understand how A/P versus L/R neuronal polarity is established in VC4 and VC5 and to determine when polarity defects first manifest in PCP mutants we monitored VC neuritogenesis in wild-type and mutant animals at specific milestones during early vulval development: the 1-cell P6.p VPC stage and following two P6.p divisions, the 2-cell and 4-cell stages [37] (Figure 3A and 3B). An early morphological marker of neurite emergence is the formation of polarized leading edge protrusions [3]. These were visualized using a soluble GFP expressed from the VC1–6 reporter transgene *cyIs3[Punc-4::GFP]*.

Neuritogenesis was followed in the vulval-proximal VC4 and VC5 neurons and VC3, a representative vulval-distal neuron. At the 1-cell P6.p stage, approximately 40–60% of VC3, VC4 and VC5 neurons displayed bipolar morphologies with anterior and posteriorly-directed protrusions. After each subsequent P6.p division, the proportion of bipolar A/P-elongated morphologies was seen to increase in VC3 neurons. In contrast, during this period, an increasing proportion of VC4 and VC5 neurons polarized growth cone protrusions exclusively toward the vulval axis of symmetry (Figure 3C). By the 4-cell P6.p stage, we found that L/R-bipolar morphology was established by bifurcation of these protrusions into two neurites that extend or squeeze laterally around the nearest VPC (Figure 3B and Figure S1). This nascent L/R polarity is maintained during the later morphogenetic VPC migrations and fusions that generate the mature vulva (Figure S1). These observations suggest that the mature L/R polarity of VC4/VC5 neurite extension is established after an earlier polarity-breaking step in which default bipolar protrusions along the A/P axis are converted into unipolar protrusions directed towards the vulval axis of symmetry and their subsequent consolidation into neurites growing laterally along the vulval epithelium.

In *vang-1* and *prkl-1* mutants, the morphology of VC4 and VC5 neurons resembled wild-type at first, but by the 4-cell P6.p stage displayed significantly more (p<0.05) A/P-elongated bipolar morphologies compared to wild-type (Figure 3D and 3E). Interestingly, in mutant animals, the proportion of VC4 and VC5 neurons that displayed misoriented protrusions at this early stage appeared to be less than the proportion that displayed ectopic neurites in adults. For example, in *prkl-1* mutants, approximately 50% of VC4 and VC5 neurons displayed protrusions directed away from P6.p vulval cells at the 4-cell stage compared to approximately 95% with A/P-directed neurites in adults. A subsequent comparison of polarity phenotypes at early, mid-L4, and adult revealed a gradual loss in polarized neuronal morphology as manifested by an increase in the proportion of A/P-directed ectopic neurites with developmental time (Figure 3F). These findings suggest that the predominant role of PCP-like signaling in VC4 and VC5 is to maintain neuronal morphology by blocking neurite formation away from vulval guidepost targets during and/or following a bipolar to unipolar reorientation of neuronal polarity along the A/P axis. Because many VC4 and VC5 neurons polarize normally during neuritogenesis in PCP mutants, the initial switch from a default A/P bipolar morphology to one oriented unidirectionally toward the vulva may not involve PCP-like signaling.

### *prkl-1* overexpression is sufficient to suppress neurite formation in VC4 and VC5

**Figure 4:**
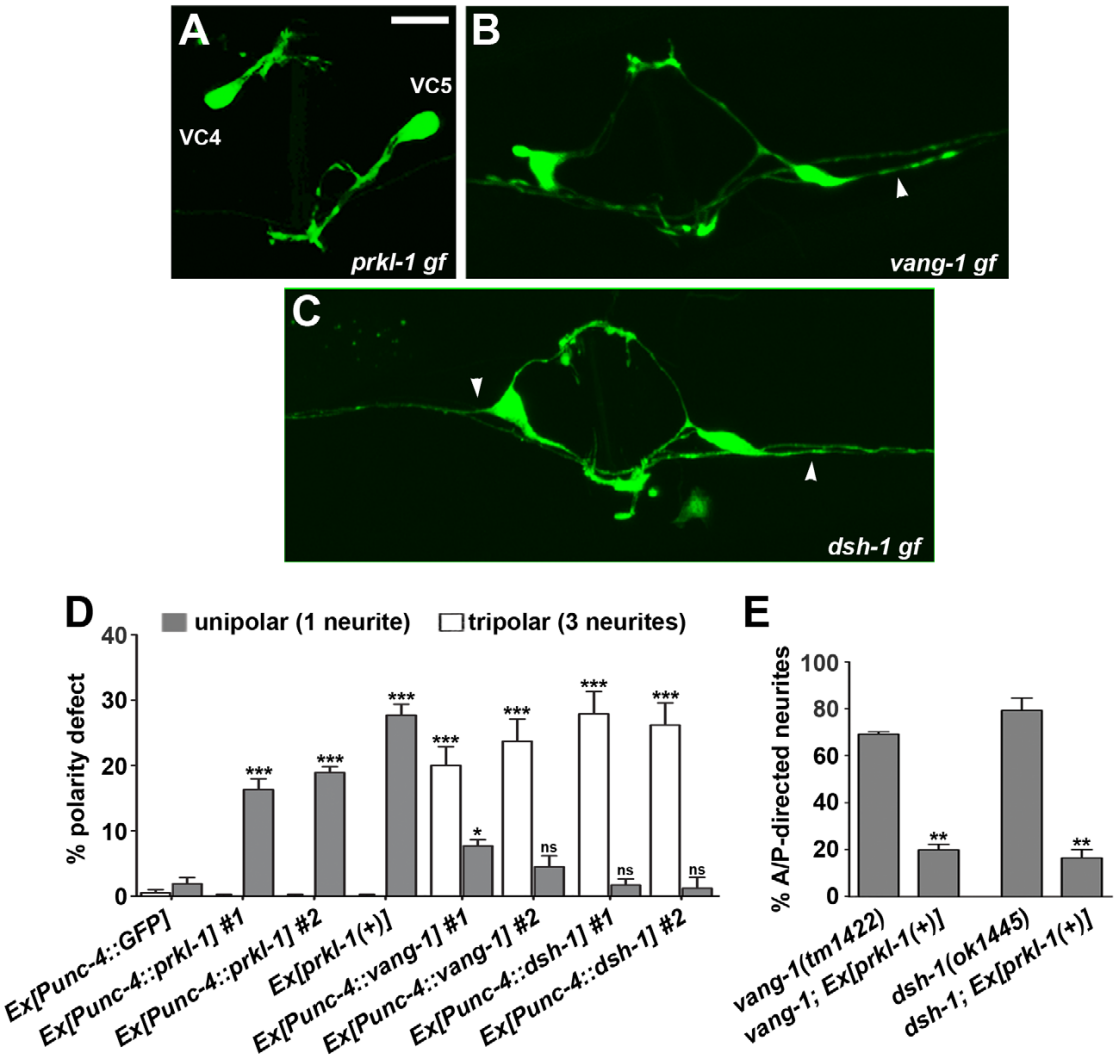
PRKL-1 is sufficient to suppress neurite formation in VC4 and VC5. (A–D) *prkl-1*, *vang-1* and *dsh-1* gain-of-function (gf) obtained by *Punc-4*-driven overexpression in wild-type animals alters the normal bipolar morphology of VC4 and VC5 neurons. *prkl-1* and to a lesser extent *vang-1* overexpression inhibits neurite formation to generate a significant increase in unipolar neurons (A and D). In contrast, *vang-1* and *dsh-1* overexpression leads to a significant increase in A/P-directed neurites (arrowheads) resulting in tripolar neurons that resemble the loss-of-function (B–D). Error bars, standard error of proportion (n>155), *p<0.01, ***p<0.0001 χ^2^. (E) A transgene expressing *prkl-1(+)* restores normal polarity in *vang-1* and *dsh-1* mutants, suggesting a downstream role during PCP signaling. Error bars, SEM, **p<0.001, t-test. VC4 and VC5 were visualized using *cyIs4*. Scale bar, 10 µm.

Loss of *vang-1*, *prkl-1*, or *dsh-1* disrupts polarity maintenance in VC4 and VC5 as shown by a bipolar to multipolar change in neuronal morphology. We therefore asked if overexpression of these components in VC4 and VC5 could suppress neurite formation to instruct a shift in polarized morphology. Alternatively, if *vang-1*, *prkl-1*, or *dsh-1* are simply required to maintain polarity once independently established, overexpression of these genes would not be expected to affect neurite number or growth. To distinguish between these possibilities, we crossed transgenic arrays carrying our *Punc-4*-driven GFP fusions, shown to support protein expression at levels that restore gene function in mutants (Figure 2E), into wild-type backgrounds. We found that 16–19% of neurons expressing *Punc-4*::*prkl-1* were unipolar compared to 4–8% expressing *vang-1*, 1–2% expressing *dsh-1*, and 2% expressing a GFP control (Figure 4A and 4D). In contrast, *Punc-4*-driven *vang-1* and *dsh-1* but not *prkl-1* resulted in tripolar neurons that resembled the loss-of-function (20–28% compared to 0% expressing *prkl-1* or GFP alone) (Figure 4B–4D). This finding is consistent with previous reports showing that, in some cases, both loss and hyperactivation of PCP proteins lead to similar polarity phenotypes [38]. Remarkably, excluding the complete loss or addition of a neurite (defined as a projection of at least 3 VC cell body diameters in length), enforced *vang-1*, *prkl-1*, or *dsh-1* expression, like the loss-of-function mutants, did not result in L/R pathfinding defects along the vulval epithelium or disorganized neuronal morphology consistent with a specific disruption in neurite formation and not general growth cone motility or neuronal homeostasis.

These findings suggest that PRKL-1 activity but not VANG-1 or DSH-1 is sufficient to suppress neurite formation in VC4 and VC5. This notion is supported by the ability of a genomic *prkl-1(+)* transgene, which results in a 30% unipolar phenotype when expressed in a wild-type background, to suppress the tripolar phenotype in *vang-1* and *dsh-1* mutants (Figure 4E). Together, these findings are consistent with a role for PCP-like signaling, and in particular, PRKL-1, in maintaining polarized neuronal morphology by actively suppressing inappropriate neurite growth rather than a more permissive ‘locking in’ role once polarity has been established.

### Localization of VANG-1 and PRKL-1-GFP fusions during neuritogenesis

Asymmetric membrane distributions of core PCP proteins are an important feature of PCP signaling in epithelial cells [36]. Likewise, polarized protein distributions in VC4 and VC5 may provide some insight into how neurite formation along the A/P axis is suppressed. We therefore examined the distribution of VANG-1 and PRKL-1 GFP fusions in VC4 and VC5 during the late L3 4-cell (P6.pxx) stage when polarity defects first manifest. GFP fusions were expressed from genome integrated versions of the extrachromosomal *Punc-4* transgenes used for rescue and overexpression studies. The ability of these fusions to restore normal polarity in PCP mutants suggest that they are able to substitute for endogenous protein function and therefore act as reasonable surrogates from which to infer endogenous localization patterns.

**Figure 5:**
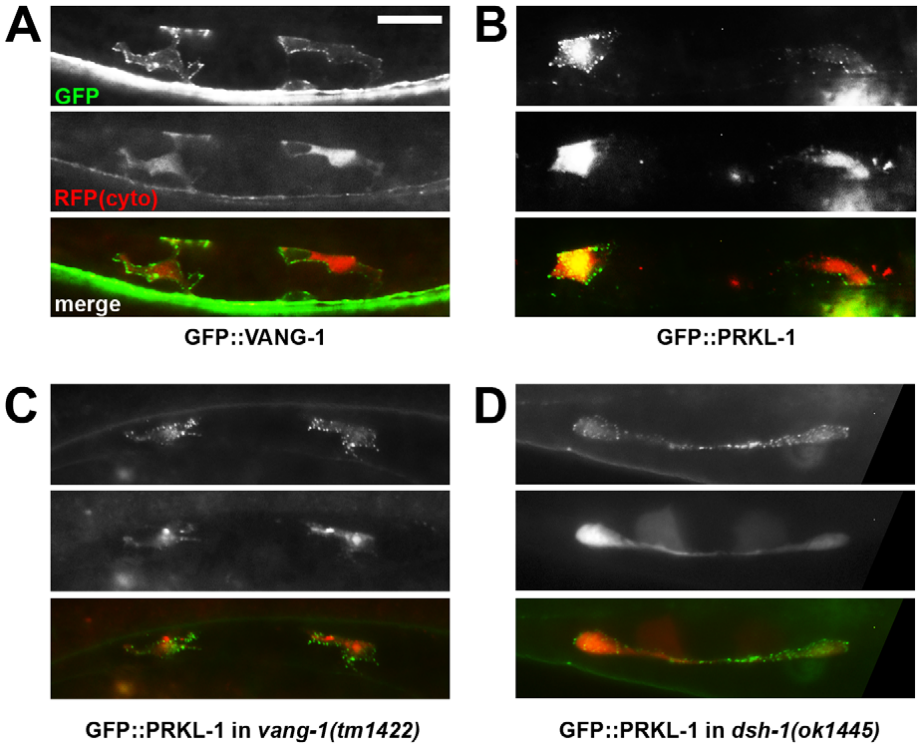
PRKL-1 and VANG-1-GFP fusions in VC neurons are localized to the plasma membrane. (A–D) Representative images of GFP-tagged VANG-1 and PRKL-1 in VC4 and VC5 neurons (ventral views) during late L3 (4-cell stage, see Figure 3). GFP-fusion proteins were expressed transgenically from *Punc-4* promoters. Soluble RFP from a *zyIs1[Plin11::RFP]* reporter background was used to label the cytoplasm (cyto) of VC neurons. GFP::VANG-1 and GFP::PRKL-1 localize in a punctate pattern on the plasma membrane. PRKL-1 recruitment to the plasma membrane was not lost in *vang-1* (C) or *dsh-1* (D) mutant backgrounds. Scale bar, 10 µm.

We found that both GFP::VANG-1 and GFP::PRKL-1 were localized symmetrically in punctate patterns on the plasma membrane of VC4 and VC5 (Figure 5A and 5B). Patterns varied from a uniform distribution along the entire membrane, including lamellipodial and neurite-like protrusions, to individual or patches of puncta distributed, in most neurons (n>20 neurons examined per fusion), without apparent polarization. These localization patterns were substantially unchanged when fusion proteins were expressed in various mutant backgrounds (n>20 neurons examined per background) (Figure 5C and 5D). Notably, PRKL-1 localization to the membrane did not appear to be appreciably affected in a *vang-1(lf)* background. This contrasts with findings in *Drosophila* cells where Van Gogh has been shown to recruit Prickle to the cell membrane during PCP activation [39], [40]. However, it is consistent with our observation that *prkl-1* overexpression can restore normal VC polarity in *vang-1* and *dsh-1* mutants suggesting that elevated PRKL-1 activity is sufficient to bypass the requirement for VANG-1 or DSH-1 during polarity signaling. Although we cannot exclude a recruitment role for VANG-1 under physiological conditions, in this situation, PRKL-1 membrane insertion may be mediated through its C-terminal CAAX farnesylation signal or its PET domain, which has recently been shown to be sufficient for membrane localization [41]. These findings suggest that polarized membrane distributions of VANG-1 or PRKL-1 do not predict sites of neurite formation or suppression; however, because asymmetries could be masked by non-physiological expression levels from transgenic arrays, a more definitive understanding requires an examination of endogenous protein distributions. It is important to note however, that polarized protein distributions are not a prominent feature of other PCP-like processes such as muscle fibre elongation and may thus reflect divergent mechanisms used to coordinate polarized activity among many cells in an epithelial sheet or tissue compared to more loosely packed or individual cells [11], [42].

### *prkl-1* overexpression in the vulval-distal VC6 neuron blocks neurite growth

**Figure 6:**
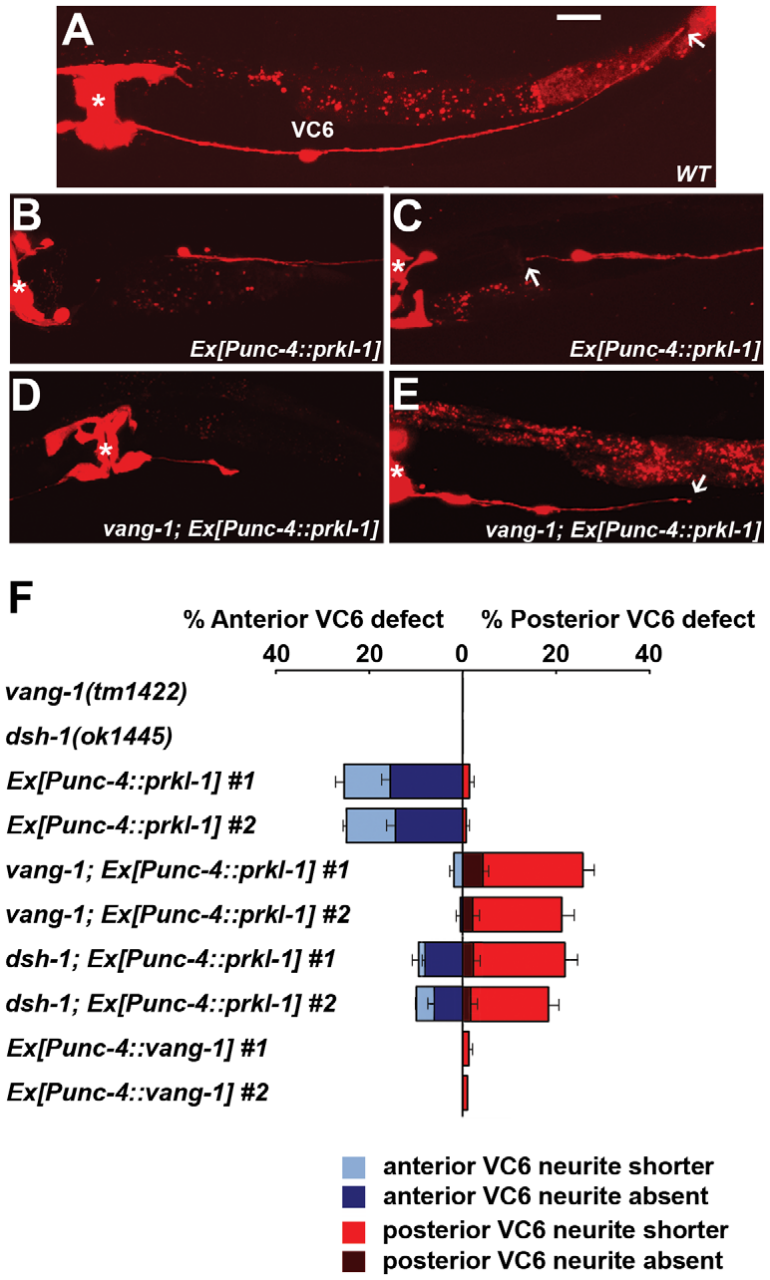
PRKL-1 overexpression in the vulval-distal VC6 neuron inhibits neurite growth. (A) The vulval-distal VC6 neuron projects an anteriorly-directed neurite that terminates at the vulva (asterisk) and a posteriorly-directed neurite that terminates in the tail region (arrow) (See also Figure 1A). (B–F) Overexpression of *prkl-1* in VC6 preferentially suppresses anterior neurite growth resulting in a specific loss (B) or premature termination (arrow in C) of the anterior neurite. However, in a *vang-1(lf)* background, posterior neurite growth is suppressed resulting in a specific loss (D) or premature termination (arrow in E) of the posterior neurite. In a *dsh-1(lf)* background, *prkl-1* overexpression suppresses posterior and to a lesser extent anterior neurite growth. (F) Quantification of *prkl-1* overexpression-induced VC6 neurite growth defects in various genetic backgrounds. All lines carry *cyIs4 and zyIs1[Plin-11::RFP]*. Error bars represent SEM, n = 3 (with at least 50 neurons examined per experiment). Scale bar, 10 µm.

The distinct phenotypes generated by a *prkl-1* loss (too many neurites) and gain-of-function (too few neurites) and the finding that PRKL-1 is sufficient to suppress neurite formation in VC4 and VC5 suggests a key role for PRKL-1 in organizing VC polarity along the A/P axis. If correct, *prkl-1* overexpression in VC1–3 or VC6 should act to suppress neurite growth and thereby reorient polarity along the A/P axis. To test this, we again utilized transgenic arrays carrying *Punc-4*::*prkl-1* to drive gene expression in all VC neurons continuously beginning in L3 prior to neurite extension. These arrays were crossed into wild-type or PCP mutant backgrounds containing *cyIs4[Pcat-1::GFP]* and *zyIs1[Plin11::RFP]* to visualize VC4 and VC5 (GFP) and VC1–6 (RFP) respectively. For this experiment we could only unambiguously assess the polarity of VC6 as VC1–3 neurites display extensive overlap along their lengths and therefore cannot be individually visualized (Figure 6A).

We found that *prkl-1* overexpression in VC6 led to a dramatic asymmetric neuronal morphology in which the anteriorly-directed neurite was either absent (14–16%) or shortened (11%) compared to the posteriorly-directed neurite (0% absent and 1–3% shortened) (Figure 6B, 6C, and 6F). Strikingly, in a *vang-1(lf)* background, *prkl-1* overexpression resulted in the reverse phenotype in which the posteriorly-directed neurite was either absent (2–4%) or shortened (19–21%) compared to the anteriorly-directed neurite (1% absent and 2% shortened) (Figure 6D, 6E, and 6F). Polarity was unaffected when *vang-1* was overexpressed in VC6 (Figure 6F). In a *dsh-1(lf)* background, *prkl-1* overexpression also affected posterior neurites (2–3% absent, 17–20% shortened) more severely than anterior neurites (6–8% absent, 1–4% shortened) (Figure 6F). These findings suggest that PRKL-1 is sufficient to inhibit neurite growth in VC6 and thereby shape neuronal morphology, but in a manner that involves VANG-1 and DSH-1 to orient inhibition along the A/P axis. Moreover, the ability of elevated levels of PRKL-1 to polarize VC6 is suggestive of an instructive role and further supports the notion that polarity maintenance in VC4 and VC5 involves a mechanism that actively blocks neurite emergence. Interestingly, PRKL-1-induced anterior neurite growth defects in VC6 appear to be only partially suppressed in a *dsh-1(lf)* background. This result may be explained if DSH-1 also acts downstream of PRKL-1 or normally antagonizes PRKL-1 activity.

### PRKL-1 acts persistently to maintain neuronal polarity

A striking feature of *vang-1*, *prkl-1*, and *dsh-1* mutants is the large proportion of VC4 and VC5 neurons that undergo normal morphological polarization early, but fail to maintain polarity during development and in the adult (Figure 3F). This situation may arise due to a transient requirement for these components before or during neuritogenesis which then indirectly maintains neuronal polarity at later stages of development. Alternatively, these components may be required persistently during development to actively maintain polarized morphology. To distinguish between these possibilities, we utilized a temporally-inducible RNAi approach to inactivate *prkl-1* after the establishment of normal VC4 and VC5 polarity. We reasoned that ectopic neurites would only be observed if PRKL-1 was required persistently to maintain polarity.

**Figure 7:**
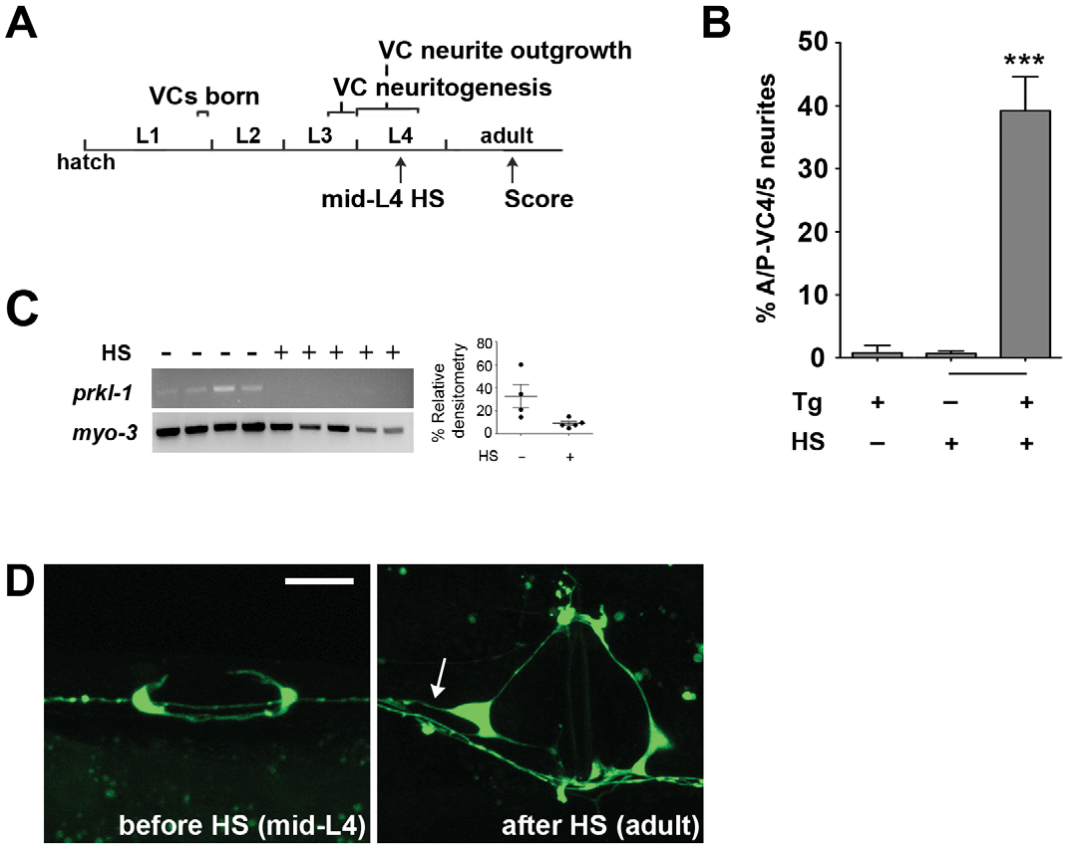
PRKL-1 is required persistently to inhibit neurite growth. (A) Timeline depicting a heat shock (HS) inducible RNAi approach to inactivate *prkl-1* following normal polarity acquisition in VC4 and VC5. (B) *prkl-1* RNAi at the mid-L4 stage when VC4 and VC5 display normal polarized morphology resulted in a significant increase in A/P-directed supernumerary neurites in the adult compared to non-HS or non-transgene (Tg) bearing animals. Error bars represent SEM. ***p<0.001, t-test. (C) RNAi depletion of *prkl-1* transcripts was verified by single worm RT-PCR in control and HS animals. Relative densitometry of *prkl-1* mRNA was normalized to control *myo-3* levels. (D) The same worm (Tg+) imaged prior to HS at mid-L4 displaying normal polarization and after HS-induced-*prkl-1* RNAi showing an ectopic VC4 neurite (arrow). VC neurons visualized using *cyIs4*. Scale bar, 10 µm.

Temporal control of *prkl-1* knock-down was achieved using an extrachromosomal transgene bearing sense and anti-sense *prkl-1* sequences under the control of the *hsp16-2* heat shock-inducible promoter [43]. This approach allows *prkl-1* RNAi to be temporally induced in transgenic animals by a heat-shock that triggers the co-expression of *prkl-1* sense and antisense transcripts throughout the animal. We found that *prkl-1* RNAi induction at the mid-L4 stage, after VC4 and VC5 neurons had undergone normal polarization and begun laterally-directed neurite extension along the vulva, resulted in the emergence of new A/P-directed neurites (∼40%) in adult animals compared to non-transgene heat shocked (∼1%) or transgene-containing non-heat shocked (∼1%) controls (Figure 7). These findings strongly support the notion that active PCP-like signaling or at least PRKL-1 activity is required persistently during development to maintain neuronal polarity.

## Discussion

**Figure 8:**
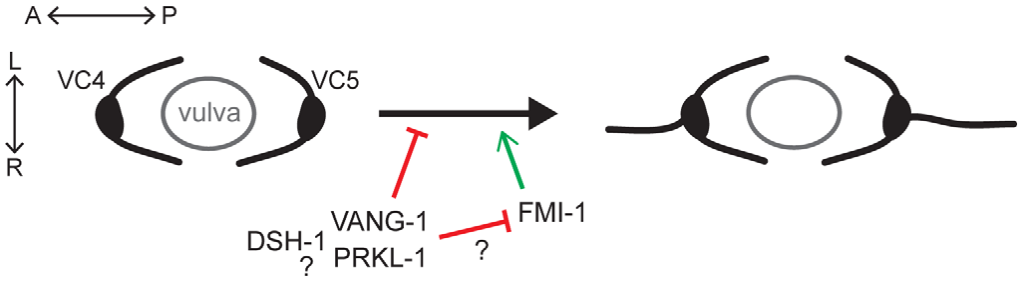
A PCP-like pathway maintains neuronal polarity by blocking ectopic A/P-directed neurite emergence in VC4 and VC5 neurons. During or following polarization of VC4 and VC5 neurite growth laterally (L/R) along the vulval epithelium, VANG-1, PRKL-1 and DSH-1 are required to maintain polarized morphology by blocking neurite emergence along the A/P axis. Genetic interactions suggest that *vang-1* and *prkl-1* act in the same genetic pathway while *dsh-1* may act in the same or parallel pathways. Loss of FMI-1 does not affect neurite emergence in a wild-type background but suppresses neurite emergence promoted by loss of VANG-1 and PRKL-1, suggesting the possibility of an inhibitory interaction between VANG-1-PRKL-1 and FMI-1 pathways.

Planar polarity refers to the polarization of cells along a specific axis of a two-dimensional plane. This definition is consistent with a process that ensures that the polarity of initial neurite emergence is restricted to a specific tissue axis. VC neurons, which appear to be morphologically similar during early larval stages, undergo a symmetry-breaking event during neuritogenesis which ultimately results in the bidirectional extension of VC1–3 and VC6 neurites along the A/P axis and VC4 and VC5 neurites laterally along the L/R axis of the developing vulva, an intermediate target path during organ innervation. We show that a PCP-like pathway in VC4 and VC5 contributes to the maintenance of this differential polarity by suppressing neurite formation along the A/P axis after normal polarization toward vulval guidepost cells (Figure 8). Loss of the core PCP genes *vang-1*/Van Gogh, *prkl-1*/Prickle, or *dsh-1*/Dishevelled results in the extension of VC4 and VC5 neurites along both the L/R organ and A/P body axes.

During late L3, prior to neurite extension, we found that VC4 and VC5 undergo a transition from a unipolar or bipolar morphology with anterior and/or posteriorly-directed leading edge protrusions into an exclusively unipolar one directed along the A/P axis towards the vulval center axis. L/R polarity is subsequently established after bifurcation of this polarized leading edge and lateral extension along vulval epithelial cells. For the most part, this earlier morphogenetic remodeling in the A/P plane, presumably due to vulval-derived polarization cues, proceeds normally but fails to be maintained in PCP mutants, giving rise to supernumerary neurites. These changes in leading edge formation during VC neuritogenesis evoke comparisons to PCP-dependent polarized cytoskeletal changes in migrating mesenchyme [17], [44], [45]. During this process, PCP disturbance results in lamellipodia that are randomized or not aligned with the normal axis of polarity causing a disruption in intercalation or migratory movements [17], [44], [46]. In worms, PCP gene loss does not lead to randomized lamellipodial protrusions but likely renders VC4 and VC5 responsive to default A/P neuritogenic or guidance cues which promote neurite growth along the A/P axis. Although the default polarity cues for VC neurons have yet to be identified, they are inferred from cell ablation studies that show VC4 and VC5 display VC1–3 and VC6-like A/P bidirectional polarity in vulva-less animals [23].

We found that *vang-1* and *dsh-1* act autonomously and non-autonomously to regulate VC polarity. In contrast, *prkl-1* is required exclusively in VC neurons. Several findings position PRKL-1 as a key regulator of polarity signaling in VC4 and VC5. First, *prkl-1* polarity defects (supernumerary VC4 and VC5 neurites) are more severe than those of *vang-1* or *dsh-1* at all stages examined. Second, *prkl-1* overexpression but not *vang-1* or *dsh-1* is sufficient to suppress neurite formation in VC4 and VC5 and restore normal polarity in *vang-1* and *dsh-1* mutants. Indeed, while *vang-1* and *dsh-1* display similar loss and gain-of-function phenotypes, loss and gain of *prkl-1* result in distinct and opposite effects on VC4 and VC5 polarity (gain or loss of a neurite respectively). Third, *prkl-1* but not *vang-1* overexpression in the vulval-distal VC6 neuron inhibits neurite growth to generate a bipolar to unipolar morphology change along the A/P axis that resembles the transition in VC4 and VC5 polarity towards VPCs during neuritogenesis. Strikingly, except for the addition or loss of a neurite, neither loss nor gain-of-functions in *vang-1*, *prkl-1*, or *dsh-1* affect other aspects of VC4 and VC5 wiring such as axon guidance along the vulval epithelium or terminal arborization, suggesting highly specific roles in regulating nascent neurite emergence.

Superficially, the fact that enforced *prkl-1* expression is sufficient to suppress neurite growth in the absence of *vang-1* and *dsh-1* and genetic interactions that place *vang-1* and *prkl-1* in the same genetic pathway are consistent with a downstream role for PRKL-1 during at least some aspects of neuronal polarity signaling. However, while PRKL-1 is sufficient to inhibit neurite growth in a *vang-1* and *dsh-1*-independent manner, the orientation of neurite inhibition along the A/P axis in VC6 is influenced by *vang-1* and *dsh-1* activity, suggesting that genetic interactions among these genes are likely more complex than a simple linear relationship. The finding that PRKL-1-GFP expression in VC neurons displayed a non-polarized cortical distribution in both wild-type and *vang-1(lf)* and *dsh-1(lf)* backgrounds argues against a simple explanation where VANG-1 and/or DSH-1 instruct PRKL-1 localization. Furthermore, although we did not investigate it further in this study, the possibility that the mature L/R-polarity of VC4 and VC5 may be specified through the partially redundant activities of a VANG-1-PRKL-1 pathway and a second DSH-1-containing pathway provides an additional level of complexity to polarity signaling in VC neurons.

In the *Drosophila* wing and thorax, the transmembrane proteins Frizzled, Van Gogh, and Flamingo interact directly across cell boundaries to propagate polarity information and thereby align the polarity of neighboring cells [12], [36], [47], [48]. The autonomous and non-autonomous activities of *vang-1* and *dsh-1* suggest that cell-cell interactions may also be involved in polarizing VC4 and VC5. *vang-1* and *dsh-1* expression in vulval cells is consistent with non-autonomous activity residing in the vulval epithelial cells that act as guidepost cells during organ innervation. Of the six VC neurons, only VC4 and VC5 are in continuous contact with their intermediate vulval cell targets throughout the period of neuritogenesis and neurite extension. Therefore, it is reasonable to speculate that these contacts contribute to multiple aspects of VC4 and VC5 identity, including the polarity of neurite emergence. Given this caveat, the finding in VC6 that the orientation of PRKL-1-induced neurite inhibition along the A/P axis is *vang-1* and *dsh-1*-dependent is consistent with the notion that VANG-1 and DSH-1 in VC4 and VC5 may act to align the output of PCP-like polarity signaling (neurite inhibition) to a specific directional vector (away from the vulva). Furthermore, since *vang-1*, *prkl-1*, and *dsh-1* are expressed in all VC neurons, vulval cell-VC communication, which may be absent in VC6, may play an important role in differentially activating and orienting neurite inhibition in VC4 and VC5.

It is not known if any combination of the four worm Fz genes act in VC polarity given the confounding effects of multiple Fz loss on vulval cell specification and polarity. Loss of *fmi-1*, the sole worm Flamingo gene, does not affect VC morphology in a wild-type background but suppresses ectopic neurite extension promoted by loss of *vang-1* and *prkl-1*. This contrasts with the similar phenotypes displayed by Van Gogh, Prickle, and Flamingo mutants on planar polarized epithelia in flies and mammals [11]–[13], suggesting that core PCP components may be utilized differently to affect polarity control in VC neurons. Interestingly, the finding from cell-specific rescue studies that epithelial derived VANG-1 can repolarize VC4 and VC5 neurons that are presumably *vang-1(lf)* suggests that a transmembrane protein on VC neurons capable of receiving polarity information remains to be identified.

At present, the PCP-like mechanism that maintains neuronal polarity by blocking neurite emergence along one directional vector but not another is not known. An attractive possibility is that PCP-like signaling acts to silence or override autonomous VC responses to default A/P guidance cues in order to limit responses to vulval-derived ones. Such switches in neuronal responses are a prominent feature of growth cone navigation at intermediate target sites where growth cones often encounter multiple guidance cues [49], [50]. We propose a model in which PCP-like proximal interactions between vulval guidepost cells and VC4 and VC5 activate a PRKL-1-dependent effector pathway in VC4 and VC5 that acts persistently to maintain morphological polarization laterally along the vulval epithelium by actively blocking neurite emergence along the orthogonal A/P axis. This model is supported by PRKL-1 overexpression in VC6 which may mimic PCP-like signaling activation, presumably not normally encountered in a vulval-distal VC neuron, and thereby suppress neurite growth along the A/P axis.

Future studies will be required to elucidate the molecular and cellular interactions involving PCP pathway components and how these interactions ultimately lead to cytoskeletal rearrangements that inhibit nascent growth cone formation. RhoA and its associated kinase ROCK are key effectors driving PCP signaling-dependent cytoskeletal changes in migrating mesenchyme [17], [44], [45]. Given that Rho and ROCK are also known to inhibit neurite growth and promote growth cone retraction in cultured neurons and *in vivo* [51], [52], it will be interesting to determine if these molecules also regulate neurite growth inhibition in VC neurons. A better understanding of how polarity control in VC neurons is achieved will also require the future elucidation of both the vulval-derived and default A/P neuritogenic or guidance cues that promote VC neurite outgrowth.

The recent discovery that extracellular guidance cues such as netrins and slits specify the site and direction of initial neurite emergence *in vivo* provided key insight into our understanding of neuronal polarity. Our study provides evidence that the maintenance of neuronal polarity *in vivo* also involves mechanisms that specifically inhibit inappropriate neurite formation along specific directional vectors that are otherwise permissive for neurite growth. These findings suggest that PCP-related signaling in higher organisms may also be involved in blocking extraneous neurite formation in developing or mature neurons that may be exposed to multiple neuritogenic or polarity cues.

## Materials and Methods

### Mutants and reporter transgenes

Worms were maintained at 20°C unless otherwise specified on *E. coli*-seeded nematode growth medium plates as described (*26*). The N2 strain was used as wild-type. The following alleles were used: LGX: *vang-1(zy2, tm1422)*, *lin-18(e620)*, *bar-1(ga80)*. LGI: *lin-44(n1792)*, *lin-17(n677)*, *mig-1(n687)*, *mom-5(or57)*. LGII: *dsh-1(ok1445)*, *mig-5(tm2639)*, *cam-1(gm122)*. LGIV: *prkl-1(zy11, ok3182)*, *egl-20(n585)*. LGV: *fmi-1(tm306)*, *cfz-2(ok1201)*. VC4 and VC5 were visualized with *cyIs1[Pcat-1::GFP]*(X) or *cyIs4[Pcat-1::GFP]*(V). VC1–6 were visualized with *cyIs3[Punc-4::GFP]* or *zyIs1[Plin-11::RFP]*.

### Genomic constructs

The Gene CATCHR yeast homologous recombination method [53] was used as described to generate GFP translational fusions of VANG-1, PRKL-1, and DSH-1 while maintaining exon-intron genomic structure. Briefly, ∼9.9 kb of *vang-1* (5′-gctctgtagagg…aaatcgagagta-3′), ∼15 kb of *prkl-1* (5′-tgcattcttcgg…ttcgtaatgccc-3′), and ∼15.8 kb of *dsh-1* (5′-gatcaatcgtgg…tttgcttctgcc-3′) containing genomic regions were recombined from YAC strains Y7110, Y40D6, and Y54E1 (gifts from A. Coulson), respectively. A GFP cassette was then inserted in-frame following the ATG start codons of *vang-1*, *prkl-1* (isoform A), and *dsh-1* (isoform C) to generate *genomic vang-1(+)*, *genomic prkl-1(+)*, and *genomic dsh-1(+)*, respectively. These constructs were injected, using standard methods (*28*), at 5 ng µl^−1^, 1 ng µl^−1^ and 10 ng µl^−1^ respectively along with 40 ng µl^−1^ of a *Podr-1::dsRed* co-transformation marker into their respective *vang-1(tm1422)*, *prkl-1(zy11)*, or *dsh-1(ok1445)* mutant backgrounds to generate the extrachromosomal arrays named *[vang-1(+)]*, *[prkl-1(+)]*, and *[dsh-1(+)]*, respectively.

### Transcriptional reporters

3 kb of *vang-1* upstream sequence was PCR-amplified from wild-type genomic DNA using primers 5′-tatgtcgactttctgggttcgtcttgagttac and 5′-ttactgcagttgatacgacatgttccacctg and inserted into pPD95.77 (a gift from A. Fire) to generate the *Pvang-1::GFP*. Similarly, 4.3 kb upstream of *dsh-1* (isoform C) was amplified using 5′-cctgtcgacgatcaatcgtggagcacatc and 5′ctggatccgtttggagcatttaatgacg to generate *Pdsh-1::GFP*. A PCR overlap extension protocol was used to make *Pprkl-1::GFP* by fusing a 9.2 kb *prkl-1* genomic region (5′-accagaatttcc…gatgttctactt-3′) ending in exon 3 in-frame to the GFP-*unc-54* 3′UTR cassette in pPD95.77. 4 kb of *lin-11* promoter was PCR-amplified using primers 5′atactgcagcccgactaaatccgacaattccg and 5′ttaggtacctgagaagggagtaaaaggaggag from genomic DNA, and inserted into pPD95.77. *Plin-11::RFP* was generated from this clone by swapping the GFP cassette with RFP. *Pvang-1::GFP* and *Pdsh-1::GFP* were individually injected at 40 ng µl^−1^ with 20 ng µl^−1^ of *Plin11::RFP* to label VC1–6 and 40 ng µl^−1^ of *Podr-1::dsRed* as a co-transformation marker into wild-type worms to generate *[Pvang-1::GFP; Plin-11::RFP]* and *[Pdsh-1::GFP; Plin-11::RFP]* transgenes. *Pprkl-1::GFP* was each injected at 30 ng µl^−1^ with 40 ng µl^−1^ of *Podr-1::dsRed* into wild-type worms. *Plin-11::RFP* was injected at 20 ng µl^−1^ with 50 ng µl^−1^ of pRF4(*rol-6gf*) and integrated using UV irradiation (UVP CL-1000 cross-linker, 254 nm, 3.25×10^4^ µJ cm^−2^) to generate *zyIs1*.

### Cell-specific expression

*vang-1* and *dsh-1* cDNAs were PCR-amplified from yk211g1 and yk291a11 (isoform C) templates (gifts from Y. Kohara), respectively. A *prkl-1* cDNA (isoform A) was obtained by RT-PCR using N2 RNA extracts. *unc-4*, *cat-1*, *ajm-1*, and *col-10* promoters were PCR-amplified (primer sequences available upon request) from previously described plasmids [26]. A *vang-1* cDNA, amplified using primers 5′cgcggatccatgtcgtatcaagataacaggaaac and 5′-tgctctagaacaaatcaaactgccgactcattgc, was inserted into pSK (Stratagene). Into this vector, we inserted a SacI-digested *vang-1* genomic fragment containing two introns and the 3′UTR PCR-amplified from genomic DNA using primers 5′aacaaccagatggactcactgaccg and 5′aacgagctcccgagactttttgtgtaatccaac. A PCR-amplified GFP cassette from pPD95.77 was then inserted in-frame into the BamHI site immediately upstream of the *vang-1* ATG start to generate *GFP::vang-1*. Cell-specific promoters were inserted immediately upstream of *GFP::vang-1* to generate *Punc-4::GFP::vang-1*, *Pcat-1::GFP::vang-1*, and *Pajm-1::GFP::vang-1*, respectively. A *prkl-1* cDNA, amplified using primers 5′-catctagaatgagcgaacgaattcgccgtc and 5′caggatcctcaagatactgtacatctggaac, was inserted into XbaI/BamHI of a GFP-less pPD95.77 vector. A GFP cassette from pPD95.77 was then inserted in-frame into SalI/XbaI upstream of the *prkl-1* ATG start to generate *GFP::prkl-1*. Cell-specific promoters were inserted upstream of *GFP::prkl-1* to generate *Punc-4::GFP::prkl-1*, *Pcat-1::GFP::prkl-1*, and *Pcol-10::GFP::prkl-1*, respectively. *dsh-1* cDNA was amplified using primers 5′tatctgcagatggccgagtctccacctcc and 5′-ctacatcccgggacatactcgtatctttgtcc and inserted into PstI/SmaI pPD95.77 to generate *dsh-1::GFP*. Cell-specific promoters were inserted into *dsh-1::GFP* to generate *Punc-4::dsh-1::GFP*, *Pcat-1::dsh-1::GFP*, and *Pcol-10::dsh-1::GFP* respectively. All constructs were injected at 10–50 ng µl^−1^ with 40 ng µl^−1^ of *Podr-1::dsRed* into *cyIs4* wild-type or their respective *vang-1(tm1422)*, *prkl-1(zy11)*, or *dsh-1(ok1445)* mutant backgrounds to generate the following transgenes: *[Punc-4::GFP::vang-1]*, *[Pcat-1::GFP::vang-1]*, *[Punc-4::GFP::prkl-1]*, *[Pcat-1::GFP::prkl-1]*, *[Pcol-10::GFP::prkl-1]*, *[Punc-4::dsh-1::GFP]*, *[Pcat-1::dsh-1::GFP]*. All experiments were performed using at least two independent transgenic lines per construct. Extrachromosomal *[Punc-4::GFP::vang-1]* and *[Punc-4::GFP::prkl-1]* transgenes for localization studies were integrated using UV irradiation as described above.

### Phenotype quantification

Worms were immobilized with 10 mM levamisole (Sigma) and imaged using an AxioplanII/Apotome or LSM510 confocal microscope and Axiovision software. Worms were staged with respect to vulval developmental milestones visualized using differential interference contrast (DIC) microscopy. See Figure S1 for images of developmental stages used in this study. Supernumerary VC4 and VC5 neurites, visualized with *cyIs4*, were scored if they were at least equal to the length of a VC cell soma in early-L4 and mid-L4 stage animals (∼5 µm) or a wild-type VC4/5 neurite (∼15 µm) in adults. The orientation of VC cell protrusions, visualized with *cyIs3*, during P6.p VPC (1-cell) and P6.p daughter (2-cell) and granddaughter (4-cell) L3 stages were determined from Apotome/Axiocam images captured sequentially with fluorescence and DIC optics. Protrusions were defined as any spike-like or ruffle-shaped extension from the cell soma. An A/P bipolar orientation was scored if a VC soma displayed at least one anteriorly and one posteriorly-directed protrusion. Unipolar orientations were scored if a VC soma displayed at least one protrusion directed either anteriorly or posteriorly. VC6 overexpression: Neurites were scored as shortened if an anterior-directed neurite failed to extend all the way to the vulva or if the posterior-directed neurite was less than approximately 60% normal length. Normally, the posterior VC6 neurite is approximately twice the length of the anterior neurite (see Figure 6A). In *vang-1* and *dsh-1* mutants, only VC6 neurons in which VC6 processes (labeled with RFP) could be distinguished from any overlapping supernumerary VC5 processes (labeled with GFP and RFP) were scored for defects. In wild-type animals, 17% (n = 225) of VC5 cell bodies are displaced one to two cell body lengths posterior to the vulval epithelium (for example, see VC5 in Figure 1D and 1E). Therefore, to further avoid ambiguities caused by overlapping VC5 neurites, only animals in which the VC5 cell body was located immediately adjacent to the vulval epithelium were scored for VC6 defects (for example, see Figure 1B and 1C).

### Inducible RNAi

Modifications were made to a previously described transgene-driven and heat shock-inducible RNAi protocol [43], [54]. A 1.1 kb fragment from a *prkl-1* cDNA (not including start codon) was PCR-amplified using primers 5′attggatcctgtgctttggacgagtatgc and 5′tatggatccgctctttgtggtggttttgg and inserted in sense and antisense orientations into BamHI pPD49.78 (a gift from A. Fire) downstream of the *hsp16-2* promoter to generate *Phsp16-2::prkl-1* sense and *Phsp16-2::prkl-1* antisense. These plasmids were then co-injected at 35 ng µl^−1^ each with 40 ng µl^−1^ of *Podr-1::dsRed* into *cyIs4* worms to generate the *[Phsp16-2::prkl-1sense; Phsp16-2::prkl-1antisense]* transgene. Coexpression of *prkl-1* sense and antisense transcripts was achieved using a 2 hour 35°C heat shock on synchronized mid-L4 stage worms with and without the transgenic array. Transgene and non-transgene bearing sib animals were then scored for ectopic VC4 and VC5 neurites in adults after an incubation of 32–34 hours at 20°C. Semi-quantitative single worm RT-PCR was used to verify RNAi-depletion of *prkl-1* mRNA. Total RNA from individual synchronized worms (chosen blindly from heat shocked and control worms) was isolated using Trizol reagent (Invitrogen) following manufacturer's instructions. Single worm RNA was solubilized in DEPC-treated water and a single step of reverse transcription (using oligo dT primers) followed by PCR was performed according to manufacturer instructions (SuperScript III One-Step RT-PCR System, Invitrogen). RT-PCR was performed with gene-specific primer pairs to *prkl-1* (5′cgaattgcagctgatgctcacag and 5′gatgtaggaagctcatgagagtac) and the internal control *myo-3* (5′atgtctggaaatccagacgcattc and 5′cgtggctccaacaatagcgaagtag). Data was analyzed by measuring the area and density of the electrophoresis bands using Scion software. Changes in *prkl-1* mRNA levels before and after RNAi induction were normalized to *myo-3* levels and expressed as arbitrary densitometry units. For each primer pair, cycle times and primer concentrations were optimized to ensure linear amplification.

## Supporting Information

Figure S1 VC4 and VC5 neurite pathfinding and vulval organogenesis. (A) Representative images of VC4 and VC5 above corresponding differential interference contrast (DIC) images showing vulval development from late L3 to adult. (B) Representative images of VC4 and VC5 (green) and subsets of vulval precursor cells (blue) at late L3, early L4 and mid-L4 stages. VC4 and VC5 and vulval precursor cells were visualized with the *Punc-4::GFP* transgene *cyIs3* and the *Pegl-17::CFP* transgene *syIs59* respectively. Scale bar, 10 µm.
(TIF)

Figure S2 VANG-1, PRKL-1, and DSH-1 domain organization and identity of molecular lesions. The domain organization of VANG-1, PRKL-1 (isoform A), and DSH-1 (isoform C) are shown below exon/intron organization of transcripts. Exons are represented by boxes and introns by solid lines. Deletion alleles are indicated by red bars. Genomic/protein domain organization and deletion data are from WormBase, http://www.wormbase.org, release WS221. Transcripts were also confirmed by direct sequencing of cDNAs.
(PDF)

Figure S3 PRKL-1 alignment. (A) Domain organization of PRKL-1, human Prickle-like 2 (PK2), and *Drosophila* Prickle (PK). Prickle orthologues contain a conserved N-terminal PET domain, three LIM domains and a CAAX prenylation signal at their C-terminus. The percentage identity of PRKL-1 conserved domains to orthologues is indicated. (B) Sequence alignment of *C. elegans* PRKL-1 and orthologues. *C. elegans* PRKL-1 (NP_741435) aligned with *Drosophila* PK Isoform A (NP_724534), zebrafish PK2 (NP_899186), mouse PK2 isoform A (NP_001074615), and human PK2 (NP_942559). Sequences corresponding to the PET domain, three LIM domains, and the CAAX prenylation signal are boxed in red, yellow, and blue, respectively. Alignment, ClustalW2.
(PDF)

Figure S4 Simultaneous loss of DSH-1 and VANG-1 or PRKL-1 leads to vulval defects. (A) Quantification of vulval phenotypes in mid-L4 stage animals. *vang-1; dsh-1* and *dsh-1; prkl-1* double mutants but not single mutants display defects in vulval cell fate specification and morphology. Vulval abnormalities were quantified using Nomarski optics at the mid-L4 ‘Christmas tree’ stage and scored as vulval defective (Vul) if displaying deficits in VPC induction or VPC fusion, and multi-vulva (Muv) if more than one vulval-like invagination was observed (n = 40–71). (B and C) Representative normal (B) and Vul (C) mid-L4 stage vulvas in *vang-1(tm1422); dsh-1(ok1445)* mutants. (D) An adult *vang-1(tm1422); dsh-1(ok1445)* animal displaying a Muv phenotype. Similar Vul and Muv phenotypes are observed in *dsh-1; prkl-1* mutants. Scale bars, 20 µm.
(TIF)

Table S1 VC4/VC5 polarity defects and *cat-1* promoter activity in various genetic backgrounds.
(PDF)
