# Supplementary figures and images for: VANG-1 and PRKL-1 Cooperate to Negatively Regulate Neurite Formation in Caenorhabditis elegans
Source: PLoS Genet. 2011 Sep 1;7(9):e1002257. doi: 10.1371/journal.pgen.1002257 (PMC3164692; doi:10.1371/journal.pgen.1002257)

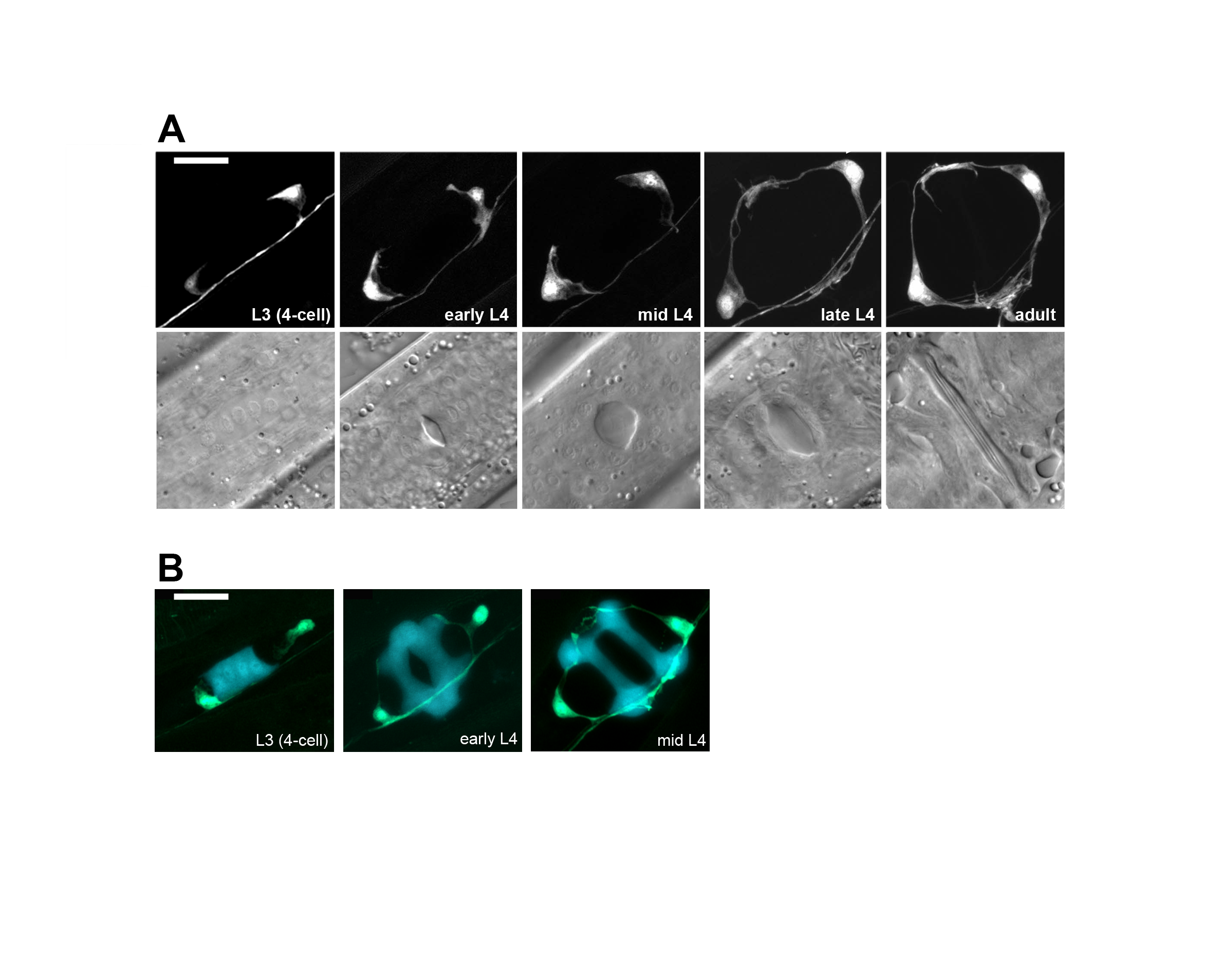

Supplement: Figure S1 — VC4 and VC5 neurite pathfinding and vulval organogenesis. (A) Representative images of VC4 and VC5 above corresponding differential interference contrast (DIC) images showing vulval development from late L3 to adult. (B) Representative images of VC4 and VC5 (green) and subsets of vulval precursor cells (blue) at late L3, early L4 and mid-L4 stages. VC4 and VC5 and vulval precursor cells were visualized with the Punc-4::GFP transgene cyIs3 and the Pegl-17::CFP transgene syIs59 respectively. Scale bar, 10 µm. (TIF) [file pgen.1002257.s001.tif]

**vang-1 genomic**

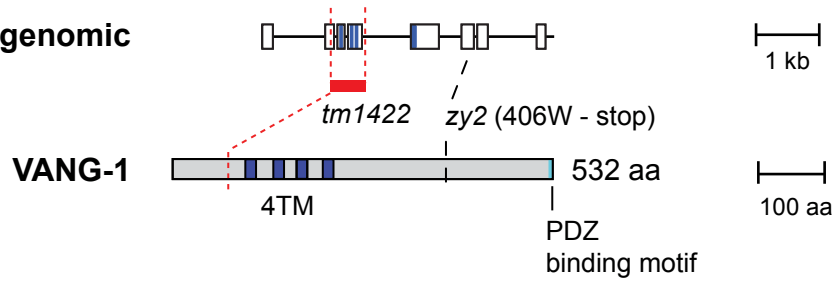

**prkl-1 genomic**

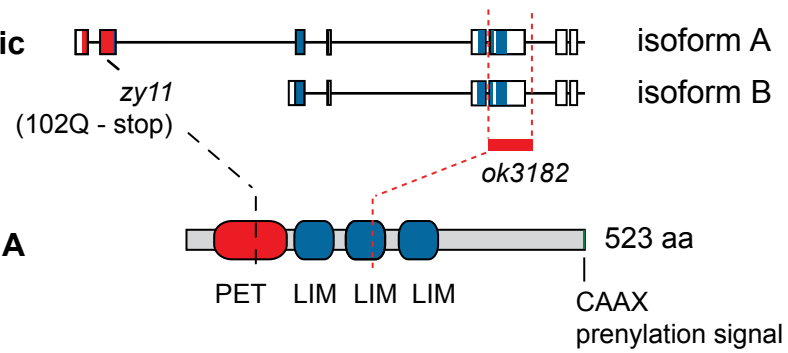

**dsh-1 genomic**

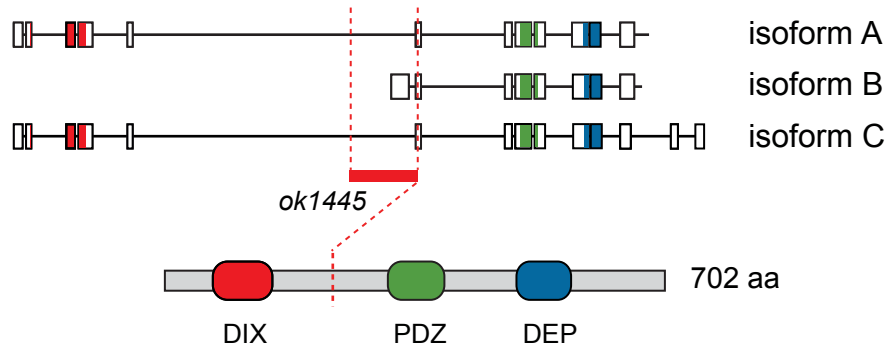

Supplement: Figure S2 — VANG-1, PRKL-1, and DSH-1 domain organization and identity of molecular lesions. The domain organization of VANG-1, PRKL-1 (isoform A), and DSH-1 (isoform C) are shown below exon/intron organization of transcripts. Exons are represented by boxes and introns by solid lines. Deletion alleles are indicated by red bars. Genomic/protein domain organization and deletion data are from WormBase, http://www.wormbase.org, release WS221. Transcripts were also confirmed by direct sequencing of cDNAs. (PDF) [file pgen.1002257.s002.pdf]

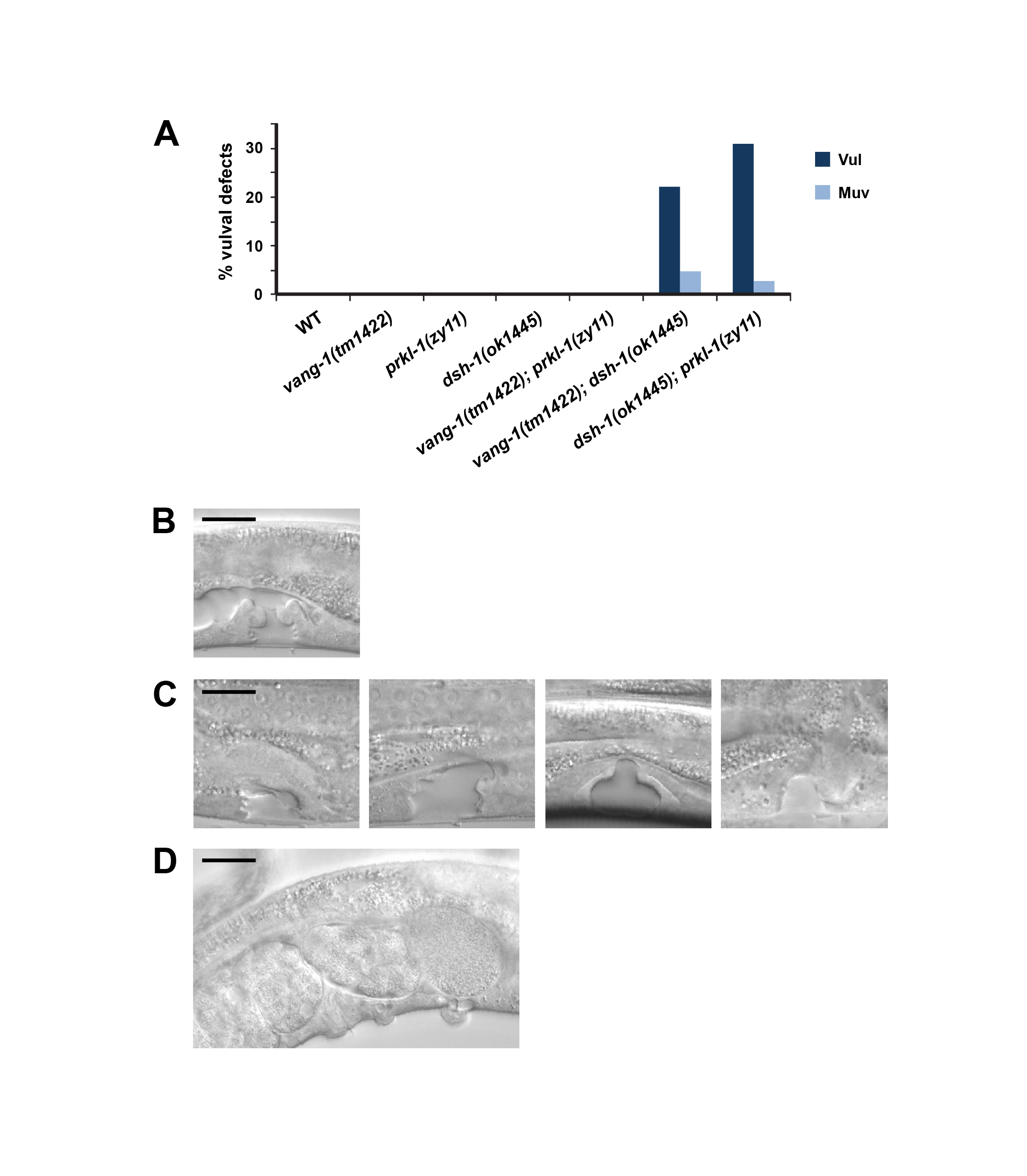

Supplement: Figure S4 — Simultaneous loss of DSH-1 and VANG-1 or PRKL-1 leads to vulval defects. (A) Quantification of vulval phenotypes in mid-L4 stage animals. vang-1; dsh-1 and dsh-1; prkl-1 double mutants but not single mutants display defects in vulval cell fate specification and morphology. Vulval abnormalities were quantified using Nomarski optics at the mid-L4 ‘Christmas tree’ stage and scored as vulval defective (Vul) if displaying deficits in VPC induction or VPC fusion, and multi-vulva (Muv) if more than one vulval-like invagination was observed (n = 40–71). (B and C) Representative normal (B) and Vul (C) mid-L4 stage vulvas in vang-1(tm1422); dsh-1(ok1445) mutants. (D) An adult vang-1(tm1422); dsh-1(ok1445) animal displaying a Muv phenotype. Similar Vul and Muv phenotypes are observed in dsh-1; prkl-1 mutants. Scale bars, 20 µm. (TIF) [file pgen.1002257.s004.tif]
