# Supplementary material for: VANG-1 and PRKL-1 Cooperate to Negatively Regulate Neurite Formation in Caenorhabditis elegans
Source: PLoS Genet. 2011 Sep 1;7(9):e1002257. doi: 10.1371/journal.pgen.1002257 (PMC3164692; doi:10.1371/journal.pgen.1002257)
Supplement: Figure S3 — PRKL-1 alignment. (A) Domain organization of PRKL-1, human Prickle-like 2 (PK2), and Drosophila Prickle (PK). Prickle orthologues contain a conserved N-terminal PET domain, three LIM domains and a CAAX prenylation signal at their C-terminus. The percentage identity of PRKL-1 conserved domains to orthologues is indicated. (B) Sequence alignment of C. elegans PRKL-1 and orthologues. C. elegans PRKL-1 (NP_741435) aligned with Drosophila PK Isoform A (NP_724534), zebrafish PK2 (NP_899186), mouse PK2 isoform A (NP_001074615), and human PK2 (NP_942559). Sequences corresponding to the PET domain, three LIM domains, and the CAAX prenylation signal are boxed in red, yellow, and blue, respectively. Alignment, ClustalW2. (PDF) [file pgen.1002257.s003.pdf]

A

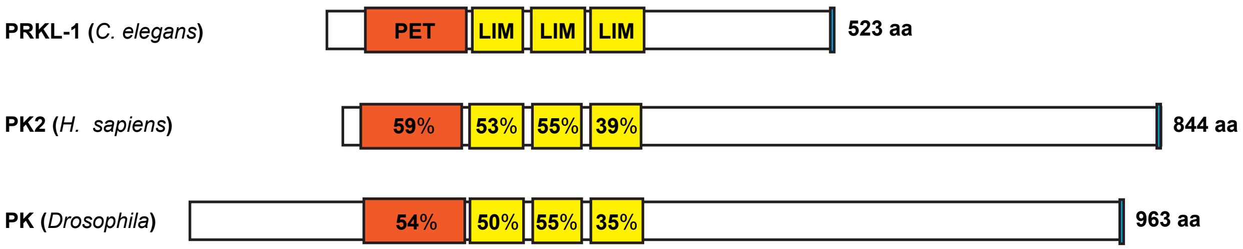

B

```
CePRKL  ------------------------------------------------------------------------------------------------------------------------
Dm PK   MDTPNQMPVELERPISRTPLTQISYLQKIPTLPRHFSPSGQGLATPPALGSGGMGLPSSSSASALYAAQAAAGILPTSPLPLQRHQQYLPPHHQQHPGAGMGPGPGSGAAAGPPLGPQYS 120
Dr PK2  ------------------------------------------------------------------------------------------------------------------------
Mm PK2  ---------------------------------------------------------------------------------------------------------MFSRSSRKRLSRRSL 15
Hs PK2  ------------------------------------------------------------------------------------------------------------------------

CePRKL  -------------------MSERIRRRRLEASEQFAPQLISSRRAQGGLSPASRIRIAADAHRHSTSDDDSGCALDEYAWVPSGLKPNMVHAYFACLPENKVPFIGSAGEKWRQRQSRYQ 101
Dm PK    PGCSANPKYSNAQLPPPPHHHHQLSPALSTPSPPSLLHHPAGGTSSASAHAPFLGGPHMDMQRQSHSDDDSGCALEEYTWVPPGLRPDQVRLYFSQIPDDKVPYVNSPGEQYRVRQLLHQ 240
Dr PK2   ---------------------------------------------MPLEMEKTVTKLMYDFQRNSTSDDDSGCALEEYAWVPPGLKPEQVHQYYSSLPEDKVPYVNSPGEKYRIKQLLHQ 75
Mm PK2  TGLGRIERGQPCNACGDQCPGFALHKWRKICLHCKCPQEEHMVTVMPLEMEKTISKLMFDFQRSSTSDDDSGCALEEYAWVPPGLKPEQVHQYYSCLPEEKVPYVNSAGEKLRIKQLLHQ 135
Hs PK2  -----------------------------------------MVTVMPLEMEKTISKLMFDFQRNSTSDDDSGCALEEYAWVPPGLKPEQVHQYYSCLPEEKVPYVNSPGEKLRIKQLLHQ 79

CePRKL  LPPQDSDVRYCEDLNAEEADTLRMFERTRKTECLGSGVVQYAPFDTK---CEKCPKRLEEGEISVMAARTGK--RYHPSCFRCQTCDVLLVDLIYFAHDNQIYCGRHHAEQVKPRCAKCD 216
Dm PK    LPPHDNEVRYCHSLTDEERKELRLFSTQRKRDALGRGNVRQLMSARP---CDGCDDLISTGDIAVFATRLGPNASWHPACFACSVCRELLVDLIYFHRDGRMYCGRHHAETLKPRCSACD 357
Dr PK2   LPPHDNEVRFCNSLDDEEKRELKLFSNQRKRENLGRGNVRPFPVTMTGAICEQCGGQINGGDIAVFASRAGHGVCWHPQCFVCSMCDELLVDLIYFYQDGKIFCGRHHAERLKPRCSACD 195
Mm PK2  LPPHDNEVRYCNSLDEEEKRELKLFSNQRKRENLGRGNVRPFPVTMTGAICEQCGGQIKGGDIAVFASRAGHGICWHPPCFVCTVCNELLVDLIYFYQDGKIYCGRHHAECLKPRCAACD 255
Hs PK2  LPPHDNEVRYCNSLDEEEKRELKLFSSQRKRENLGRGNVRPFPVTMTGAICEQCGGQINGGDIAVFASRAGHGVCWHPPCFVCTVCNELLVDLIYFYQDGKIYCGRHHAECLKPRCAACD 199

CePRKL  EVIFGDECLEAEGRSWHFHHFQCAQCNDVLADQKYMQRANKPVCLKCFHSSSSTFSCTTCRLSFSSDTPHMSQGDLHWHASAECFCCCVCSKNLLGVKYSRVGESLFCGYQTCGGEDEEL 336
Dm PK    EIILADECTEAEGRAWHMNHFACHECDKQLGGQRYIMREGKPYCLHCFDAMFAEY-CDYCGEAIGVDQGQMSHDGQHWHATDECFSCNTCRCSLLGRAFLPRRGAIYCSIACSKGEPPTP 476
Dr PK2   EIILADECTEAEGRHWHMKHFCCFECETVLGGQRYIMKEGRPYCCTCFESLYAEY-CDSCGEHIGIDQGQMTYDGQHWHATEACFSCARCKKSLLGRPFLPKQGQIFCSRACSVGDEQNG 314
Mm PK2  EIIFADECTEAEGRHWHMRHFCCFECETVLGGQRYIMKEGRPYCCHCFESLYAEY-CDTCAQHIGIDQGQMTYDGQHWHATETCFCCAHCKKSLLGRPFLPKQGQIFCSRACSAGEDPNG 374
Hs PK2  EIIFADECTEAEGRHWHMKHFCCFECETVLGGQRYIMKEGRPYCCHCFESLYAEY-CDTCAQHIGIDQGQMTYDGQHWHATETCFCCAHCKKSLLGRPFLPKQGQIFCSRACSAGEDPNG 318

CePRKL  LD--EDRLGSPHRKVTQKSTKVVRIPASPRVAPRHPHVIQQNLTTTMTIQKPSVVIQNQRPKPPQRAP---------------------------------------------------- 402
Dm PK    SDSSGTGMYTTPTPPTQRVRPHPQAPLPARIPSSHASSSPPMSPQQQQQHQATFNQAMYQMQSQQMEAAGGLVDQSKSYAASDSDAG------------------VVKDLEHGGHMGGGD 578
Dr PK2   SDSSDSAFQSARSREARRSSSKS-NKSSTGGSGGDGHQSGKASAMRFSADVDPLSLQMDLLSLSSQTPSLNRESPSWKTQAEMFGYEGRNE--LSTNPLHLLSQCNIRTSYSSN-LSPGH 430
Mm PK2  SDSSDSAFQNARAKESRRSAKIGKNKGKTEEAMLNQHSQLQVSSNRLSADVDPLSVQMDLLSLSSQTPSLNRD-PIWRSREEPFHYGNKMEQNQSQSPLQLLSQCNIRTSYSPGGQGAGA 493
Hs PK2  SDSSDSAFQNARAKESRRSAKIGKNKGKTEEPMLNQHSQLQVSSNRLSADVDPLSLQMDMLSLSSQTPSLNRD-PIWRSREEPYHYGNKMEQNQTQSPLQLLSQCNIRTSYSPGGQGAGA 437

CePRKL  ------------------------------------------------------------------------------------------------------PPPPSENIYETVLPCSSN 420
Dm PK    LTDFSGGRASSTSQNLSPLNSPGDFQPHFLPKPMELQRDGVYNFNEMSSNLDAAWSAKPTNSYHLQRQLLENPHTASMPELAGKLVAPPAHMQHLSQLHAVSSHQFQQHEYADILHPPPP 698
Dr PK2   LSDPRPKEPASSKRPPVSALKGKSLNENWFQQGPEDYYPPALRTQASFNEVPHNSFMDKR-SVSLHVFQREKEKD---VGPQMSRSRNPISALGFSEQLTPLEQTPRGSMESLALSNATG 546
Mm PK2  QPDMWAKHFSNPKRSSSMALKGHGG--SFIQECREDYYPGRLMSQESYSDMSSQSFNETRGSIPVPKYEEEEEEEEEGGISTQQCRPRRPLSSLKYTEDMTPTEQTPRGSMESLALSNATG 611
Hs PK2  QPEMWGKHFSNPKRSSSLAMTGHAG--SFIKECREDYYPGRLRSQESYSDMSSQSFSETRGSIQVPKYEEEEEEEE-GGLSTQQCRTRHPISSLKYTEDMTPTEQTPRGSMESLALSNATG 554

CePRKL  NSPNFDKKYSHELP--------------------------TSPNHHNYYSKTPNNLLTGYPEMDG------------------------------------------------------- 459
Dm PK    PPGEIPELPTPNLSVASTALPPELMGSPTHSAGDRSLNTPMSTQSASHAPPHPVSILSGASSSSPMSGEPAKKKGVRFEGIPDTLPRSRSYSG----------NGAGTSGGGERERDRDK 808
Dr PK2   TSADGGSKRQEHLSRFSMPDLSKDSGMNVSEK-SNMGTLNSSVQFHSSESLRSLNSAQPYLELEAPVQVRFPVQYPREPSGINALPPGFAYQEEDRVSLVSNANNARLPPMSERTRRRSI 665
Mm PK2  LSAEGGAKRQEHLSRFSMPDLSKDSGMNVSEKLSNMGTLNSSMQFRSAESVRSLLSAQQYQEMEGNLHQLSNPLGYRDLQSHGRMHQSFDFDGGIASSKLPGQEGVHIQPMSERTRRRTT 731
Hs PK2  LSADGGAKRQEHLSRFSMPDLSKDSGMNVSEKLSNMGTLNSSMQFRSAESVRSLLSAQQYQEMEGNLHQLSNPIGYRDLQSHGRMHQSFDFDGGMAGSKLPGQEGVRIQPMSERTRRRAT 674

CePRKL  -------------------------------------------------------------------------------------------------------------YSTSSSSDSDD 470
Dm PK    DKEGGGRHGHGHSSRRRRRRKSSSS-------------SSHHRSGSGHRSHSTTRADTYAPAQPLSSSYQGPPSVLQAANLVHESPSRQQREREREREREESEESDVCSTCSSSSSSS- 913
Dr PK2   DHEEPRRRHHHHRSRRSRRSRSDNALNLVAER--RPALK-RSQLHAREDYDQFPLPRGSRDTYGSGRLRQQPFRPCPRTTSDLTLQNPALHRHQGPYSWDQYDYDDDWCSTCSSSSESED 782
Mm PK2  SRDD-NRRFRPHRSRRSRRSRSDNALHLASEREVIARLKERPPLRAREDYDQFMRQRSFQESLGQG-SRRDLYSQCPRTVSDLALQNAFGER------WGPYFTEYDWCSTCSSSSESDN 843
Hs PK2  SRDD-NRRFRPHRSRRSRRSRSDNALHLASEREAISRLKDRPPLRAREDYDQFMRQRSFQESMGHG-SRRDLYGQCPRTVSDLALQNAFGDR------WGPYFAEYDWCSTCSSSSESDN 786

CePRKL  EQLYISN-IMAAASLSRVPAKSSSRKSKK----NEPMMMSGGGVRMAKKKKSSRCTVS 523
Dm PK    EDYMMMYQLPQRRHYGGVRVSYVPN--------DALAYDRKRKPSELGGDKDKNCIIS 963
Dr PK2   EGYFLGEPIPRPIQLRHMTSEELLRKYNSSGLGASGQFGSRGQLHMRKRRKSKNCIIS 840
Mm PK2  EGYFLGEPIPQPARLRYVTSDELLHKYSSYGVPKSSTLGGRGQLHSRKRQKSKNCIIS 901
Hs PK2  EGYFLGEPIPQPARLRYVTSDELLHKYSSYGLPKSSTLGGRGQLHSRKRQKSKNCIIS 844
```
