# Supplementary material for: VANG-1 and PRKL-1 Cooperate to Negatively Regulate Neurite Formation in Caenorhabditis elegans
Source: PLoS Genet. 2011 Sep 1;7(9):e1002257. doi: 10.1371/journal.pgen.1002257 (PMC3164692; doi:10.1371/journal.pgen.1002257)
Supplement: Table S1 — VC4/VC5 polarity defects and cat-1 promoter activity in various genetic backgrounds. (PDF) [file pgen.1002257.s005.pdf]

Table S1. VC4/VC5 polarity defects and *cat-1* promoter activity in various genetic backgrounds.

Genotype*	VC4 % A/P-oriented neurites	VC4 % *Pcat1::GFP* positive	VC4 n	VC5 % A/P-oriented neurites	VC5 % *Pcat1::GFP* positive	VC5 n	# of worms
Wild type	1	100	548	0.6	99.8	547	548
**Wnt**							
lin-44(n1792)	nd	0	-	nd	0	-	>100
egl-20(n585)	2.2	97.9	46	0	100	47	47
**Van Gogh**							
vang-1(tm1422)	66.6	97.7	543	68.7	97.3	541	556
**Prickle**							
prkl-1(zy11)	95.9	97.8	217	96.3	97.3	216	222
**Frizzled**							
cfz-2(ok1220)	1.9	99	103	1.9	99	103	104
mig-1(n687)	3	99	147	2	99	145	148
mig-1(n687); cfz-2(ok1220)	1	96	146	1	97	149	157
lin-17(n677)	nd	0	-	nd	0	-	>100
*mom-5(or57)***	0	95.8	23	0	100	24	24
**Dishevelled**							
dsh-1(ok1445)	76.8	68.9	155	64	90.2	203	225
dsh-2(or302)†	nd	nd	-	nd	nd	-	-
mig-5(tm2639)	nd	0	-	nd	0	-	>100
**Flamingo**							
fmi-1(tm306)	0	100	128	0	100	128	128
**Ryk**							
lin-18(e620) ‡	5.5	82.6	90	0	55	60	109
**Ror**							
cam-1(gm122) §	0	88.7	63	3	92.9	66	71
**β-catenin**							
bar-1(ga80)	nd	0	-	nd	0	-	>100

* All lines contain *cyIs4[Pcat-1::GFP]* except *fmi-1(tm306)* which contains *cyIs1[Pcat-1::GFP]*. *Pcat-1* driven GFP expression was lost in 100% of VC4 and VC5 neurons in *lin-44*/Wnt, *lin-17*/Fz, *mig-5*/Dsh, and *bar-1*/β-catenin mutants. *cat-1* promoter activity in PDE and head sensory neurons was not affected. nd, not determined.

** Maternally rescued *mom-5(or57)* worms from *dpy-5(e61) mom-5(or57)/hT2; +/hT2[bli-4(e937) let-?(h661)]* were scored.

† *or302* is embryonic lethal.

‡ *lin-18(e620)* mutants display enhanced *Pcat-1::GFP* expression in HSN neurons. VC4 and VC5 were therefore scored in *lin-18(e620); egl-1(n987)* animals.

§ Some *cam-1* mutants display mispositioned VC neurons or additional *Pcat-1::GFP* positive neurons flanking vulva.
